# Genetic drivers of heterogeneity in type 2 diabetes pathophysiology

**DOI:** 10.1038/s41586-024-07019-6

**Published:** 2024-02-19

**Authors:** Ken Suzuki, Konstantinos Hatzikotoulas, Lorraine Southam, Henry J. Taylor, Xianyong Yin, Kim M. Lorenz, Ravi Mandla, Alicia Huerta-Chagoya, Giorgio E. M. Melloni, Stavroula Kanoni, Nigel W. Rayner, Ozvan Bocher, Ana Luiza Arruda, Kyuto Sonehara, Shinichi Namba, Simon S. K. Lee, Michael H. Preuss, Lauren E. Petty, Philip Schroeder, Brett Vanderwerff, Mart Kals, Fiona Bragg, Kuang Lin, Xiuqing Guo, Weihua Zhang, Jie Yao, Young Jin Kim, Mariaelisa Graff, Fumihiko Takeuchi, Jana Nano, Amel Lamri, Masahiro Nakatochi, Sanghoon Moon, Robert A. Scott, James P. Cook, Jung-Jin Lee, Ian Pan, Daniel Taliun, Esteban J. Parra, Jin-Fang Chai, Lawrence F. Bielak, Yasuharu Tabara, Yang Hai, Gudmar Thorleifsson, Niels Grarup, Tamar Sofer, Matthias Wuttke, Chloé Sarnowski, Christian Gieger, Darryl Nousome, Stella Trompet, Soo-Heon Kwak, Jirong Long, Meng Sun, Lin Tong, Wei-Min Chen, Suraj S. Nongmaithem, Raymond Noordam, Victor J. Y. Lim, Claudia H. T. Tam, Yoonjung Yoonie Joo, Chien-Hsiun Chen, Laura M. Raffield, Bram Peter Prins, Aude Nicolas, Lisa R. Yanek, Guanjie Chen, Jennifer A. Brody, Edmond Kabagambe, Ping An, Anny H. Xiang, Hyeok Sun Choi, Brian E. Cade, Jingyi Tan, K. Alaine Broadaway, Alice Williamson, Zoha Kamali, Jinrui Cui, Manonanthini Thangam, Linda S. Adair, Adebowale Adeyemo, Carlos A. Aguilar-Salinas, Tarunveer S. Ahluwalia, Sonia S. Anand, Alain Bertoni, Jette Bork-Jensen, Ivan Brandslund, Thomas A. Buchanan, Charles F. Burant, Adam S. Butterworth, Mickaël Canouil, Juliana C. N. Chan, Li-Ching Chang, Miao-Li Chee, Ji Chen, Shyh-Huei Chen, Yuan-Tsong Chen, Zhengming Chen, Lee-Ming Chuang, Mary Cushman, John Danesh, Swapan K. Das, H. Janaka de Silva, George Dedoussis, Latchezar Dimitrov, Ayo P. Doumatey, Shufa Du, Qing Duan, Kai-Uwe Eckardt, Leslie S. Emery, Daniel S. Evans, Michele K. Evans, Krista Fischer, James S. Floyd, Ian Ford, Oscar H. Franco, Timothy M. Frayling, Barry I. Freedman, Pauline Genter, Hertzel C. Gerstein, Vilmantas Giedraitis, Clicerio González-Villalpando, Maria Elena González-Villalpando, Penny Gordon-Larsen, Myron Gross, Lindsay A. Guare, Sophie Hackinger, Liisa Hakaste, Sohee Han, Andrew T. Hattersley, Christian Herder, Momoko Horikoshi, Annie-Green Howard, Willa Hsueh, Mengna Huang, Wei Huang, Yi-Jen Hung, Mi Yeong Hwang, Chii-Min Hwu, Sahoko Ichihara, Mohammad Arfan Ikram, Martin Ingelsson, Md. Tariqul Islam, Masato Isono, Hye-Mi Jang, Farzana Jasmine, Guozhi Jiang, Jost B. Jonas, Torben Jørgensen, Frederick K. Kamanu, Fouad R. Kandeel, Anuradhani Kasturiratne, Tomohiro Katsuya, Varinderpal Kaur, Takahisa Kawaguchi, Jacob M. Keaton, Abel N. Kho, Chiea-Chuen Khor, Muhammad G. Kibriya, Duk-Hwan Kim, Florian Kronenberg, Johanna Kuusisto, Kristi Läll, Leslie A. Lange, Kyung Min Lee, Myung-Shik Lee, Nanette R. Lee, Aaron Leong, Liming Li, Yun Li, Ruifang Li-Gao, Symen Ligthart, Cecilia M. Lindgren, Allan Linneberg, Ching-Ti Liu, Jianjun Liu, Adam E. Locke, Tin Louie, Jian’an Luan, Andrea O. Luk, Xi Luo, Jun Lv, Julie A. Lynch, Valeriya Lyssenko, Shiro Maeda, Vasiliki Mamakou, Sohail Rafik Mansuri, Koichi Matsuda, Thomas Meitinger, Olle Melander, Andres Metspalu, Huan Mo, Andrew D. Morris, Filipe A. Moura, Jerry L. Nadler, Michael A. Nalls, Uma Nayak, Ioanna Ntalla, Yukinori Okada, Lorena Orozco, Sanjay R. Patel, Snehal Patil, Pei Pei, Mark A. Pereira, Annette Peters, Fraser J. Pirie, Hannah G. Polikowsky, Bianca Porneala, Gauri Prasad, Laura J. Rasmussen-Torvik, Alexander P. Reiner, Michael Roden, Rebecca Rohde, Katheryn Roll, Charumathi Sabanayagam, Kevin Sandow, Alagu Sankareswaran, Naveed Sattar, Sebastian Schönherr, Mohammad Shahriar, Botong Shen, Jinxiu Shi, Dong Mun Shin, Nobuhiro Shojima, Jennifer A. Smith, Wing Yee So, Alena Stančáková, Valgerdur Steinthorsdottir, Adrienne M. Stilp, Konstantin Strauch, Kent D. Taylor, Barbara Thorand, Unnur Thorsteinsdottir, Brian Tomlinson, Tam C. Tran, Fuu-Jen Tsai, Jaakko Tuomilehto, Teresa Tusie-Luna, Miriam S. Udler, Adan Valladares-Salgado, Rob M. van Dam, Jan B. van Klinken, Rohit Varma, Niels Wacher-Rodarte, Eleanor Wheeler, Ananda R. Wickremasinghe, Ko Willems van Dijk, Daniel R. Witte, Chittaranjan S. Yajnik, Ken Yamamoto, Kenichi Yamamoto, Kyungheon Yoon, Canqing Yu, Jian-Min Yuan, Salim Yusuf, Matthew Zawistowski, Liang Zhang, Wei Zheng, VA Million Veteran Program, Leslie J. Raffel, Michiya Igase, Eli Ipp, Susan Redline, Yoon Shin Cho, Lars Lind, Michael A. Province, Myriam Fornage, Craig L. Hanis, Erik Ingelsson, Alan B. Zonderman, Bruce M. Psaty, Ya-Xing Wang, Charles N. Rotimi, Diane M. Becker, Fumihiko Matsuda, Yongmei Liu, Mitsuhiro Yokota, Sharon L. R. Kardia, Patricia A. Peyser, James S. Pankow, James C. Engert, Amélie Bonnefond, Philippe Froguel, James G. Wilson, Wayne H. H. Sheu, Jer-Yuarn Wu, M. Geoffrey Hayes, Ronald C. W. Ma, Tien-Yin Wong, Dennis O. Mook-Kanamori, Tiinamaija Tuomi, Giriraj R. Chandak, Francis S. Collins, Dwaipayan Bharadwaj, Guillaume Paré, Michèle M. Sale, Habibul Ahsan, Ayesha A. Motala, Xiao-Ou Shu, Kyong-Soo Park, J. Wouter Jukema, Miguel Cruz, Yii-Der Ida Chen, Stephen S. Rich, Roberta McKean-Cowdin, Harald Grallert, Ching-Yu Cheng, Mohsen Ghanbari, E-Shyong Tai, Josee Dupuis, Norihiro Kato, Markku Laakso, Anna Köttgen, Woon-Puay Koh, Donald W. Bowden, Colin N. A. Palmer, Jaspal S. Kooner, Charles Kooperberg, Simin Liu, Kari E. North, Danish Saleheen, Torben Hansen, Oluf Pedersen, Nicholas J. Wareham, Juyoung Lee, Bong-Jo Kim, Iona Y. Millwood, Robin G. Walters, Kari Stefansson, Emma Ahlqvist, Mark O. Goodarzi, Karen L. Mohlke, Claudia Langenberg, Christopher A. Haiman, Ruth J. F. Loos, Jose C. Florez, Daniel J. Rader, Marylyn D. Ritchie, Sebastian Zöllner, Reedik Mägi, Nicholas A. Marston, Christian T. Ruff, David A. van Heel, Sarah Finer, Joshua C. Denny, Toshimasa Yamauchi, Takashi Kadowaki, John C. Chambers, Maggie C. Y. Ng, Xueling Sim, Jennifer E. Below, Philip S. Tsao, Kyong-Mi Chang, Mark I. McCarthy, James B. Meigs, Anubha Mahajan, Cassandra N. Spracklen, Josep M. Mercader, Michael Boehnke, Jerome I. Rotter, Marijana Vujkovic, Benjamin F. Voight, Andrew P. Morris, Eleftheria Zeggini

**Affiliations:** 1 Centre for Genetics and Genomics Versus Arthritis, Centre for Musculoskeletal Research, Division of Musculoskeletal and Dermatological Sciences, University of Manchester, Manchester, UK; 2 Department of Diabetes and Metabolic Diseases, Graduate School of Medicine, University of Tokyo, Tokyo, Japan; 3 Department of Statistical Genetics, Osaka University Graduate School of Medicine, Suita, Japan; 4 Institute of Translational Genomics, Helmholtz Zentrum München, German Research Center for Environmental Health, Neuherberg, Germany; 5 Center for Precision Health Research, National Human Genome Research Institute, National Institutes of Health, Bethesda, MD USA; 6 British Heart Foundation Cardiovascular Epidemiology Unit, Department of Public Health and Primary Care, University of Cambridge, Cambridge, UK; 7 Heart and Lung Research Institute, University of Cambridge, Cambridge, UK; 8 Department of Epidemiology, School of Public Health, Nanjing Medical University, Nanjing, China; 9 Department of Biostatistics and Center for Statistical Genetics, University of Michigan, Ann Arbor, MI USA; 10 Corporal Michael J. Crescenz VA Medical Center, Philadelphia, PA USA; 11 Department of Systems Pharmacology and Translational Therapeutics, University of Pennsylvania Perelman School of Medicine, Philadelphia, PA USA; 12 Department of Genetics, University of Pennsylvania Perelman School of Medicine, Philadelphia, PA USA; 13 Programs in Metabolism and Medical and Population Genetics, Broad Institute of Harvard and MIT, Cambridge, MA USA; 14 Diabetes Unit and Center for Genomic Medicine, Massachusetts General Hospital, Boston, MA USA; 15 TIMI Study Group, Division of Cardiovascular Medicine, Brigham and Women’s Hospital, Harvard Medical School, Boston, MA USA; 16 William Harvey Research Institute, Barts and the London School of Medicine and Dentistry, Queen Mary University of London, London, UK; 17 Graduate School of Experimental Medicine, Technical University of Munich, Munich, Germany; 18 Munich School for Data Science, Helmholtz Munich, Neuherberg, Germany; 19 Department of Genome Informatics, Graduate School of Medicine, University of Tokyo, Tokyo, Japan; 20 Integrated Frontier Research for Medical Science Division, Institute for Open and Transdisciplinary Research Initiatives, Osaka University, Suita, Japan; 21 Laboratory for Systems Genetics, RIKEN Center for Integrative Medical Sciences, Yokohama, Japan; 22 Charles Bronfman Institute for Personalized Medicine, Icahn School of Medicine at Mount Sinai, New York, NY USA; 23 Department of Medicine, Vanderbilt University Medical Center, Nashville, TN USA; 24 Estonian Genome Centre, Institute of Genomics, University of Tartu, Tartu, Estonia; 25 Nuffield Department of Population Health, University of Oxford, Oxford, UK; 26 Medical Research Council Population Health Research Unit, University of Oxford, Oxford, UK; 27 Institute for Translational Genomics and Population Sciences, Department of Pediatrics, Lundquist Institute for Biomedical Innovation at Harbor-UCLA Medical Center, Torrance, CA USA; 28 Department of Epidemiology and Biostatistics, Imperial College London, London, UK; 29 Department of Cardiology, Ealing Hospital, London NorthWest Healthcare NHS Trust, London, UK; 30 Division of Genome Science, Department of Precision Medicine, National Institute of Health, Cheongju-si, South Korea; 31 Department of Epidemiology, Gillings School of Global Public Health, University of North Carolina at Chapel Hill, Chapel Hill, NC USA; 32 Department of Gene Diagnostics and Therapeutics, Research Institute, National Center for Global Health and Medicine, Tokyo, Japan; 33 Institute of Epidemiology, Helmholtz Zentrum München, German Research Center for Environmental Health, Neuherberg, Germany; 34 Department of Medicine, McMaster University, Hamilton, Ontario Canada; 35 Population Health Research Institute, Hamilton Health Sciences and McMaster University, Hamilton, Ontario Canada; 36 Public Health Informatics Unit, Department of Integrated Health Sciences, Nagoya University Graduate School of Medicine, Nagoya, Japan; 37 MRC Epidemiology Unit, Institute of Metabolic Science, University of Cambridge School of Clinical Medicine, Cambridge, UK; 38 Department of Health Data Science, University of Liverpool, Liverpool, UK; 39 Division of Translational Medicine and Human Genetics, University of Pennsylvania, Philadelphia, PA USA; 40 Department of Epidemiology, Brown University School of Public Health, Providence, RI USA; 41 Department of Anthropology, University of Toronto at Mississauga, Mississauga, Ontario Canada; 42 Saw Swee Hock School of Public Health, National University of Singapore and National University Health System, Singapore, Singapore; 43 Department of Epidemiology, School of Public Health, University of Michigan, Ann Arbor, MI USA; 44 Center for Genomic Medicine, Kyoto University Graduate School of Medicine, Kyoto, Japan; 45 deCODE Genetics, Amgen, Reykjavik, Iceland; 46 Novo Nordisk Foundation Center for Basic Metabolic Research, Faculty of Health and Medical Sciences, University of Copenhagen, Copenhagen, Denmark; 47 Department of Biostatistics, Harvard University, Boston, MA USA; 48 Division of Sleep and Circadian Disorders, Brigham and Women’s Hospital, Boston, MA USA; 49 Department of Medicine, Harvard University, Boston, MA USA; 50 Institute of Genetic Epidemiology, Department of Data Driven Medicine, Faculty of Medicine and Medical Center, University of Freiburg, Freiburg, Germany; 51 Department of Epidemiology, Human Genetics and Environmental Sciences, University of Texas Health Science Center at Houston School of Public Health, Houston, TX USA; 52 German Center for Diabetes Research (DZD), Neuherberg, Germany; 53 Research Unit of Molecular Epidemiology, Helmholtz Zentrum München, German Research Center for Environmental Health, Neuherberg, Germany; 54 Department of Population and Public Health Sciences, Keck School of Medicine of USC, Los Angeles, CA USA; 55 Department of Cardiology, Leiden University Medical Center, Leiden, The Netherlands; 56 Section of Gerontology and Geriatrics, Department of Internal Medicine, Leiden University Medical Center, Leiden, The Netherlands; 57 Department of Internal Medicine, Seoul National University Hospital, Seoul, South Korea; 58 Division of Epidemiology, Department of Medicine, Institute for Medicine and Public Health, Vanderbilt Genetics Institute, Vanderbilt University Medical Center, Nashville, TN USA; 59 Nuffield Department of Surgical Sciences, University of Oxford, Oxford, UK; 60 Institute for Population and Precision Health (IPPH), Biological Sciences Division, University of Chicago, Chicago, IL USA; 61 Department of Public Health Sciences and Center for Public Health Genomics, University of Virginia School of Medicine, Charlottesville, VA USA; 62 Genomic Research on Complex Diseases (GRC-Group), CSIR-Centre for Cellular and Molecular Biology (CSIR-CCMB), Hyderabad, India; 63 Department of Medicine and Therapeutics, Chinese University of Hong Kong, Hong Kong, China; 64 Chinese University of Hong Kong–Shanghai Jiao Tong University Joint Research Centre in Diabetes Genomics and Precision Medicine, Chinese University of Hong Kong, Hong Kong, China; 65 Samsung Advanced Institute for Health Sciences & Technology (SAIHST), Sungkyunkwan University, Samsung Medical Center, Seoul, South Korea; 66 Department of Preventive Medicine, Northwestern University Feinberg School of Medicine, Chicago, IL USA; 67 Institute of Biomedical Sciences, Academia Sinica, Taipei, Taiwan; 68 Department of Genetics, University of North Carolina at Chapel Hill, Chapel Hill, NC USA; 69 Department of Human Genetics, Wellcome Sanger Institute, Wellcome Genome Campus, Hinxton, UK; 70 Laboratory of Neurogenetics, National Institute on Aging, National Institutes of Health, Bethesda, MD USA; 71 Department of Medicine, Johns Hopkins University School of Medicine, Baltimore, MD USA; 72 Center for Research on Genomics and Global Health, National Human Genome Research Institute, National Institutes of Health, Bethesda, MD USA; 73 Cardiovascular Health Research Unit, Department of Medicine, University of Washington, Seattle, WA USA; 74 Division of Academics, Ochsner Health, New Orleans, LA USA; 75 Division of Statistical Genomics, Washington University School of Medicine, St Louis, MO USA; 76 Department of Research and Evaluation, Division of Biostatistics Research, Kaiser Permanente of Southern California, Pasadena, CA USA; 77 Department of Biomedical Science, Hallym University, Chuncheon, South Korea; 78 Harvard Medical School, Boston, MA USA; 79 Metabolic Research Laboratories, Wellcome Trust–Medical Research Council Institute of Metabolic Science, Department of Clinical Biochemistry, University of Cambridge, Cambridge, UK; 80 Department of Epidemiology, University of Groningen, University Medical Centre Groningen, Groningen, The Netherlands; 81 Department of Bioinformatics, Isfahan University of Medical Sciences, Isfahan, Iran; 82 Department of Medicine, Division of Endocrinology, Diabetes and Metabolism, Cedars-Sinai Medical Center, Los Angeles, CA USA; 83 Lund University Diabetes Centre, Department of Clinical Sciences, Lund University, Skåne University Hospital, Malmö, Sweden; 84 Department of Nutrition, Gillings School of Global Public Health, University of North Carolina at Chapel Hill, Chapel Hill, NC USA; 85 Unidad de Investigación en Enfermedades Metabólicas and Departamento de Endocrinología y Metabolismo, Instituto Nacional de Ciencias Médicas y Nutrición Salvador Zubirán, Mexico City, Mexico; 86 Steno Diabetes Center Copenhagen, Herlev, Denmark; 87 Bioinformatics Center, Department of Biology, University of Copenhagen, Copenhagen, Denmark; 88 Department of Health Research Methods, Evidence and Impact, McMaster University, Hamilton, Ontario Canada; 89 Department of Epidemiology and Prevention, Division of Public Health Sciences, Wake Forest School of Medicine, Winston-Salem, NC USA; 90 Institute of Regional Health Research, University of Southern Denmark, Odense, Denmark; 91 Department of Clinical Biochemistry, Vejle Hospital, Vejle, Denmark; 92 Department of Medicine, Division of Endocrinology and Diabetes, Keck School of Medicine of USC, Los Angeles, CA USA; 93 Department of Internal Medicine, University of Michigan, Ann Arbor, MI USA; 94 British Heart Foundation Centre of Research Excellence, School of Clinical Medicine, Addenbrooke’s Hospital, University of Cambridge, Cambridge, UK; 95 Health Data Research UK Cambridge, Wellcome Genome Campus, University of Cambridge, Hinxton, UK; 96 National Institute for Health and Care Research (NIHR) Blood and Transplant Unit (BTRU) in Donor Health and Behaviour, Heart and Lung Research Institute, University of Cambridge, Cambridge, UK; 97 Inserm U1283, CNRS UMR 8199, European Genomic Institute for Diabetes (EGID), Institut Pasteur de Lille, Lille University Hospital, Lille, France; 98 University of Lille, Lille, France; 99 Li Ka Shing Institute of Health Sciences, Chinese University of Hong Kong, Hong Kong, China; 100 Hong Kong Institute of Diabetes and Obesity, Chinese University of Hong Kong, Hong Kong, China; 101 Singapore Eye Research Institute, Singapore National Eye Centre, Singapore, Singapore; 102 Exeter Centre of Excellence in Diabetes (ExCEeD), Exeter Medical School, University of Exeter, Exeter, UK; 103 Wellcome Sanger Institute, Wellcome Genome Campus, Hinxton, UK; 104 Department of Biostatistics and Data Science, Wake Forest School of Medicine, Winston-Salem, NC USA; 105 Division of Endocrinology and Metabolism, Department of Internal Medicine, National Taiwan University Hospital, Taipei, Taiwan; 106 Institute of Epidemiology and Preventive Medicine, National Taiwan University, Taipei, Taiwan; 107 Department of Medicine, University of Vermont, Colchester, VT USA; 108 Section of Endocrinology and Metabolism, Department of Internal Medicine, Wake Forest School of Medicine, Winston-Salem, NC USA; 109 Department of Medicine, Faculty of Medicine, University of Kelaniya, Ragama, Sri Lanka; 110 Department of Nutrition and Dietetics, Harokopio University of Athens, Athens, Greece; 111 Center for Genomics and Personalized Medicine Research, Wake Forest School of Medicine, Winston-Salem, NC USA; 112 Carolina Population Center, University of North Carolina at Chapel Hill, Chapel Hill, NC USA; 113 Department of Nephrology and Medical Intensive Care Medicine, Charité–Universitätsmedizin Berlin, Berlin, Germany; 114 Department of Nephrology and Hypertension, Friedrich-Alexander-Universität Erlangen-Nürnberg, Erlangen, Germany; 115 Department of Biostatistics, University of Washington, Seattle, WA USA; 116 California Pacific Medical Center Research Institute, San Francisco, CA USA; 117 Laboratory of Epidemiology and Population Sciences, National Institute on Aging, National Institutes of Health, Baltimore, MD USA; 118 Institute of Mathematics and Statistics, University of Tartu, Tartu, Estonia; 119 Robertson Centre for Biostatistics, University of Glasgow, Glasgow, UK; 120 Department of Epidemiology, Erasmus MC University Medical Center, Rotterdam, The Netherlands; 121 Genetics of Complex Traits, University of Exeter Medical School, University of Exeter, Exeter, UK; 122 Department of Internal Medicine, Wake Forest School of Medicine, Winston-Salem, NC USA; 123 Department of Medicine, Division of Endocrinology and Metabolism, Lundquist Research Institute at Harbor-UCLA Medical Center, Torrance, CA USA; 124 Department of Public Health and Caring Sciences, Uppsala University, Uppsala, Sweden; 125 Centro de Estudios en Diabetes, Unidad de Investigacion en Diabetes y Riesgo Cardiovascular, Centro de Investigacion en Salud Poblacional, Instituto Nacional de Salud Publica, Mexico City, Mexico; 126 Department of Laboratory Medicine and Pathology, University of Minnesota, Minneapolis, MN USA; 127 Genomics and Computational Biology Graduate Group, University of Pennsylvania Perelman School of Medicine, Philadelphia, PA USA; 128 Institute for Molecular Medicine Finland (FIMM), University of Helsinki, Helsinki, Finland; 129 Folkhalsan Research Center, Helsinki, Finland; 130 University of Exeter Medical School, University of Exeter, Exeter, UK; 131 Institute for Clinical Diabetology, German Diabetes Center, Leibniz Center for Diabetes Research at Heinrich Heine University Düsseldorf, Düsseldorf, Germany; 132 Department of Endocrinology and Diabetology, Medical Faculty and University Hospital Düsseldorf, Heinrich Heine University Düsseldorf, Düsseldorf, Germany; 133 Laboratory for Genomics of Diabetes and Metabolism, RIKEN Center for Integrative Medical Sciences, Yokohama, Japan; 134 Department of Biostatistics, Gillings School of Global Public Health, University of North Carolina at Chapel Hill, Chapel Hill, NC USA; 135 Department of Internal Medicine, Diabetes and Metabolism Research Center, Ohio State University Wexner Medical Center, Columbus, OH USA; 136 Center for Global Cardiometabolic Health, Brown University, Providence, RI USA; 137 Shanghai-MOST Key Laboratory of Health and Disease Genomics, Shanghai Institute for Biomedical and Pharmaceutical Technologies, Shanghai, China; 138 Division of Endocrine and Metabolism, Tri-Service General Hospital Songshan Branch, Taipei, Taiwan; 139 School of Medicine, National Defense Medical Center, Taipei, Taiwan; 140 Division of Genome Science, Department of Precision Medicine, National Institute of Health, Cheongju-si, Korea; 141 Section of Endocrinology and Metabolism, Department of Medicine, Taipei Veterans General Hospital, Taipei, Taiwan; 142 School of Medicine, National Yang Ming Chiao Tung University, Taipei, Taiwan; 143 Department of Environmental and Preventive Medicine, Jichi Medical University School of Medicine, Shimotsuke, Japan; 144 University of Chicago Research Bangladesh, Dhaka, Bangladesh; 145 Institute of Molecular and Clinical Ophthalmology Basel, Basel, Switzerland; 146 Center for Clinical Research and Prevention, Bispebjerg and Frederiksberg Hospital, Frederiksberg, Denmark; 147 Faculty of Health and Medical Sciences, University of Copenhagen, Copenhagen, Denmark; 148 Faculty of Medicine, Aalborg University, Aalborg, Denmark; 149 Department of Clinical Diabetes, Endocrinology and Metabolism, Department of Translational Research and Cellular Therapeutics, City of Hope, Duarte, CA USA; 150 Department of Public Health, Faculty of Medicine, University of Kelaniya, Ragama, Sri Lanka; 151 Department of Clinical Gene Therapy, Osaka University Graduate School of Medicine, Osaka, Japan; 152 Department of Geriatric and General Medicine, Graduate School of Medicine, Osaka University, Osaka, Japan; 153 Division of General Internal Medicine and Geriatrics, Department of Medicine, Northwestern University Feinberg School of Medicine, Chicago, IL USA; 154 Center for Health Information Partnerships, Institute for Public Health and Medicine, Northwestern University Feinberg School of Medicine, Chicago, IL USA; 155 Genome Institute of Singapore, Agency for Science, Technology and Research, Singapore, Singapore; 156 Department of Molecular Cell Biology, Sungkyunkwan University School of Medicine, Suwon, South Korea; 157 Institute of Genetic Epidemiology, Medical University of Innsbruck, Innsbruck, Austria; 158 Institute of Clinical Medicine, Internal Medicine, University of Eastern Finland and Kuopio University Hospital, Kuopio, Finland; 159 Department of Medicine, University of Colorado Denver, Anschutz Medical Campus, Aurora, CO USA; 160 VA Salt Lake City Health Care System, Salt Lake City, UT USA; 161 Department of Internal Medicine, University of Utah School of Medicine, Salt Lake City, UT USA; 162 Soochunhyang Institute of Medi-bio Science and Division of Endocrinology, Department of Internal Medicine, Soochunhyang University College of Medicine, Cheonan, South Korea; 163 Department of Medicine, Samsung Medical Center, Sungkyunkwan University School of Medicine, Seoul, South Korea; 164 USC-Office of Population Studies Foundation, University of San Carlos, Cebu City, Philippines; 165 Department of Medicine, Harvard Medical School, Boston, MA USA; 166 Division of General Internal Medicine, Massachusetts General Hospital, Boston, MA USA; 167 Department of Epidemiology and Biostatistics, School of Public Health, Peking University, Beijing, China; 168 Peking University Center for Public Health and Epidemic Preparedness and Response, Beijing, China; 169 Department of Clinical Epidemiology, Leiden University Medical Center, Leiden, The Netherlands; 170 Wellcome Centre for Human Genetics, Nuffield Department of Medicine, University of Oxford, Oxford, UK; 171 Program in Medical and Population Genetics, Broad Institute, Cambridge, MA USA; 172 Big Data Institute, Li Ka Shing Centre for Health Information and Discovery, University of Oxford, Oxford, UK; 173 Department of Clinical Medicine, Faculty of Health and Medical Sciences, University of Copenhagen, Copenhagen, Denmark; 174 Department of Biostatistics, Boston University School of Public Health, Boston, MA USA; 175 Department of Medicine, Yong Loo Lin School of Medicine, National University of Singapore and National University Health System, Singapore, Singapore; 176 McDonnell Genome Institute, Washington University School of Medicine, St Louis, MO USA; 177 Department of Medicine, Division of Genomics and Bioinformatics, Washington University School of Medicine, St Louis, MO USA; 178 Department of Biostatistics and Data Science, University of Texas Health Science Center at Houston School of Public Health, Houston, TX USA; 179 Department of Clinical Sciences, Diabetes and Endocrinology, Lund University Diabetes Centre, Malmö, Sweden; 180 Department of Clinical Science, Center for Diabetes Research, University of Bergen, Bergen, Norway; 181 Department of Advanced Genomic and Laboratory Medicine, Graduate School of Medicine, University of the Ryukyus, Nishihara, Japan; 182 Division of Clinical Laboratory and Blood Transfusion, University of the Ryukyus Hospital, Nishihara, Japan; 183 Dromokaiteio Psychiatric Hospital, National and Kapodistrian University of Athens, Athens, Greece; 184 Academy of Scientific and Innovative Research (AcSIR), Ghaziabad, India; 185 Computational Biology and Medical Sciences, Graduate School of Frontier Sciences, University of Tokyo, Tokyo, Japan; 186 Institute of Human Genetics, Helmholtz Zentrum München, German Research Center for Environmental Health, Neuherberg, Germany; 187 Institute of Human Genetics, Technical University Munich, Munich, Germany; 188 German Centre for Cardiovascular Research (DZHK), Partner Site Munich Heart Alliance, Munich, Germany; 189 Usher Institute to the Population Health Sciences and Informatics, University of Edinburgh, Edinburgh, UK; 190 Department of Medicine and Pharmacology, New York Medical College, Valhalla, NY USA; 191 Data Tecnica International, Glen Echo, MD USA; 192 Center for Alzheimer’s and Related Dementias, National Institutes of Health, Bethesda, MD USA; 193 Laboratory of Statistical Immunology, Immunology Frontier Research Center (WPI-IFReC), Osaka University, Suita, Japan; 194 Premium Research Institute for Human Metaverse Medicine (WPI-PRIMe), Osaka University, Suita, Japan; 195 Instituto Nacional de Medicina Genómica, Mexico City, Mexico; 196 Division of Pulmonary, Allergy, and Critical Care Medicine, Department of Medicine, University of Pittsburgh, Pittsburgh, PA USA; 197 Division of Epidemiology and Community Health, School of Public Health, University of Minnesota, Minneapolis, MN USA; 198 Institute for Medical Information Processing, Biometry and Epidemiology, Ludwig-Maximilians-Universität München, Munich, Germany; 199 Department of Diabetes and Endocrinology, Nelson R. Mandela School of Medicine, College of Health Sciences, University of KwaZulu-Natal, Durban, South Africa; 200 Academy of Scientific and Innovative Research, CSIR-Human Resource Development Campus, Ghaziabad, India; 201 Genomics and Molecular Medicine Unit, CSIR-Institute of Genomics and Integrative Biology, New Delhi, India; 202 Fred Hutchinson Cancer Research Center, Seattle, WA USA; 203 Ophthalmology and Visual Sciences Academic Clinical Program (Eye ACP), Duke-NUS Medical School, Singapore, Singapore; 204 Department of Ophthalmology, Yong Loo Lin School of Medicine, National University of Singapore and National University Health System, Singapore, Singapore; 205 School of Cardiovascular and Metabolic Health, University of Glasgow, Glasgow, UK; 206 Survey Research Center, Institute for Social Research, University of Michigan, Ann Arbor, MI USA; 207 Institute of Genetic Epidemiology, Helmholtz Zentrum Munchen, German Research Center for Environmental Health, Neuherberg, Germany; 208 Institute for Medical Biostatistics, Epidemiology, and Informatics (IMBEI), University Medical Center, Johannes Gutenberg University, Mainz, Germany; 209 Chair of Genetic Epidemiology, Institute of Medical Information Processing, Biometry, and Epidemiology, Faculty of Medicine, Ludwig-Maximilians-Universität München, Munich, Germany; 210 Faculty of Medicine, University of Iceland, Reykjavik, Iceland; 211 Faculty of Medicine, Macau University of Science and Technology, Macau, China; 212 Department of Medical Genetics and Medical Research, China Medical University Hospital, Taichung, Taiwan; 213 Population Health Unit, Finnish Institute for Health and Welfare, Helsinki, Finland; 214 National School of Public Health, Madrid, Spain; 215 Department of Public Health, University of Helsinki, Helsinki, Finland; 216 Diabetes Research Group, King Abdulaziz University, Jeddah, Saudi Arabia; 217 Unidad de Biología Molecular y Medicina Genómica, Instituto Nacional de Ciencias Médicas y Nutrición Salvador Zubirán, Mexico City, Mexico; 218 Departamento de Medicina Genómica y Toxiología Ambiental, Instituto de Investigaciones Biomédicas, UNAM, Mexico City, Mexico; 219 Unidad de Investigacion Medica en Bioquimica, Hospital de Especialidades, Centro Medico Nacional Siglo XXI, Instituto Mexicano del Seguro Social, Mexico City, Mexico; 220 Einthoven Laboratory for Experimental Vascular Medicine, Leiden University Medical Center, Leiden, The Netherlands; 221 Department of Human Genetics, Leiden University Medical Center, Leiden, The Netherlands; 222 Department of Clinical Chemistry, Laboratory of Genetic Metabolic Disease, Amsterdam University Medical Center, Amsterdam, The Netherlands; 223 Southern California Eye Institute, CHA Hollywood Presbyterian Hospital, Los Angeles, CA USA; 224 Unidad de Investigación Médica en Epidemiologia Clinica, Hospital de Especialidades, Centro Medico Nacional Siglo XXI, Instituto Mexicano del Seguro Social, Mexico City, Mexico; 225 Department of Internal Medicine, Division of Endocrinology, Leiden University Medical Center, Leiden, The Netherlands; 226 Department of Public Health, Aarhus University, Aarhus, Denmark; 227 Danish Diabetes Academy, Odense, Denmark; 228 Diabetology Research Centre, King Edward Memorial Hospital and Research Centre, Pune, India; 229 Department of Medical Biochemistry, Kurume University School of Medicine, Kurume, Japan; 230 Department of Pediatrics, Osaka University Graduate School of Medicine, Suita, Japan; 231 Division of Cancer Control and Population Sciences, UPMC Hillman Cancer Center, University of Pittsburgh, Pittsburgh, PA USA; 232 Department of Epidemiology, Graduate School of Public Health, University of Pittsburgh, Pittsburgh, PA USA; 233 Department of Pediatrics, Division of Genetic and Genomic Medicine, UCI Irvine School of Medicine, Irvine, CA USA; 234 Department of Anti-Aging Medicine, Ehime University Graduate School of Medicine, Touon, Japan; 235 Division of Pulmonary, Critical Care, and Sleep Medicine, Beth Israel Deaconess Medical Center, Boston, MA USA; 236 Department of Medical Sciences, Uppsala University, Uppsala, Sweden; 237 Institute of Molecular Medicine, University of Texas Health Science Center at Houston School of Public Health, Houston, TX USA; 238 Human Genetics Center, University of Texas Health Science Center at Houston, Houston, TX USA; 239 Department of Medicine, Stanford University School of Medicine, Stanford, CA USA; 240 Department of Medical Sciences, Molecular Epidemiology and Science for Life Laboratory, Uppsala University, Uppsala, Sweden; 241 Department of Epidemiology, University of Washington, Seattle, WA USA; 242 Department of Health Systems and Population Health, University of Washington, Seattle, WA USA; 243 Beijing Institute of Ophthalmology, Ophthalmology and Visual Sciences Key Laboratory, Beijing Tongren Hospital, Capital Medical University, Beijing, China; 244 Department of Medicine, Division of Cardiology, Duke University School of Medicine, Durham, NC USA; 245 Kurume University School of Medicine, Kurume, Japan; 246 Department of Medicine, McGill University, Montreal, Quebec Canada; 247 Department of Human Genetics, McGill University, Montreal, Quebec Canada; 248 Department of Metabolism, Digestion and Reproduction, Imperial College London, London, UK; 249 Division of Cardiovascular Medicine, Beth Israel Deaconess Medical Center, Boston, MA USA; 250 Division of Endocrinology and Metabolism, Department of Medicine, Taichung Veterans General Hospital, Taichung, Taiwan; 251 Division of Endocrinology, Metabolism and Molecular Medicine, Department of Medicine, Northwestern University Feinberg School of Medicine, Chicago, IL USA; 252 Center for Genetic Medicine, Northwestern University Feinberg School of Medicine, Chicago, IL USA; 253 Department of Anthropology, Northwestern University, Evanston, IL USA; 254 Department of Endocrinology, Helsinki University Hospital, Helsinki, Finland; 255 Science and Engineering Research Board (SERB), Department of Science and Technology, Ministry of Science and Technology, Government of India, New Delhi, India; 256 Systems Genomics Laboratory, School of Biotechnology, Jawaharlal Nehru University, New Delhi, India; 257 Department of Pathology and Molecular Medicine, McMaster University, Hamilton, Ontario Canada; 258 Department of Molecular Medicine and Biopharmaceutical Sciences, Graduate School of Convergence Science and Technology, Seoul National University, Seoul, South Korea; 259 Netherlands Heart Institute, Utrecht, The Netherlands; 260 Center for Public Health Genomics, University of Virginia School of Medicine, Charlottesville, VA USA; 261 Research Unit of Molecular Epidemiology, Helmholtz Zentrum München, German Research Center for Environmental Health, Munich, Germany; 262 Duke-NUS Medical School, Singapore, Singapore; 263 Department of Epidemiology, Biostatistics and Occupational Health, McGill University, Montreal, Quebec Canada; 264 Singapore Institute for Clinical Sciences, Agency for Science Technology and Research (A*STAR), Singapore, Singapore; 265 Healthy Longevity Translational Research Programme, Yong Loo Lin School of Medicine, National University of Singapore, Singapore, Singapore; 266 Center for Diabetes Research, Wake Forest School of Medicine, Winston-Salem, NC USA; 267 Department of Biochemistry, Wake Forest School of Medicine, Winston-Salem, NC USA; 268 Pat Macpherson Centre for Pharmacogenetics and Pharmacogenomics, University of Dundee, Dundee, UK; 269 Imperial College Healthcare NHS Trust, Imperial College London, London, UK; 270 MRC-PHE Centre for Environment and Health, Imperial College London, London, UK; 271 National Heart and Lung Institute, Imperial College London, London, UK; 272 Department of Medicine, Brown University Alpert School of Medicine, Providence, RI USA; 273 Department of Medicine, Columbia University Irving Medical Center, New York, NY USA; 274 Department of Cardiology, Columbia University Irving Medical Center, New York, NY USA; 275 Center for Non-Communicable Diseases, Karachi, Pakistan; 276 Computational Medicine, Berlin Institute of Health at Charité–Universitätsmedizin, Berlin, Germany; 277 Precision Healthcare University Research Institute, Queen Mary University of London, London, UK; 278 Department of Preventive Medicine, Keck School of Medicine of USC, Los Angeles, CA USA; 279 The Mindich Child Health and Development Institute, Ichan School of Medicine at Mount Sinai, New York, NY USA; 280 Division of Translational Medicine and Therapeutics, Department of Medicine, University of Pennsylvania Perelman School of Medicine, Philadelphia, PA USA; 281 Institute for Translational Medicine and Therapeutics, University of Pennsylvania Perelman School of Medicine, Philadelphia, PA USA; 282 Department of Pediatrics, University of Pennsylvania Perelman School of Medicine, Philadelphia, PA USA; 283 Center for Precision Medicine, University of Pennsylvania - Perelman School of Medicine, Philadelphia, PA USA; 284 Institute for Biomedical Informatics, University of Pennsylvania Perelman School of Medicine, Philadelphia, PA USA; 285 Department of Psychiatry, University of Michigan, Ann Arbor, MI USA; 286 Blizard Institute, Queen Mary University of London, London, UK; 287 Institute for Population Health Sciences, Barts and the London School of Medicine and Dentistry, Queen Mary University of London, London, UK; 288 All of Us Research Program, National Institutes of Health, Bethesda, MD USA; 289 Toranomon Hospital, Tokyo, Japan; 290 Lee Kong Chian School of Medicine, Nanyang Technological University, Singapore, Singapore; 291 Vanderbilt Genetics Institute, Division of Genetic Medicine, Vanderbilt University Medical Center, Nashville, TN USA; 292 VA Palo Alto Health Care System, Palo Alto, CA USA; 293 Stanford Cardiovascular Institute, Stanford University School of Medicine, Stanford, CA USA; 294 Department of Medicine, University of Pennsylvania Perelman School of Medicine, Philadelphia, PA USA; 295 Oxford Centre for Diabetes, Endocrinology and Metabolism, Radcliffe Department of Medicine, University of Oxford, Oxford, UK; 296 Oxford NIHR Biomedical Research Centre, Churchill Hospital, Oxford University Hospitals NHS Foundation Trust, Oxford, UK; 297 Department of Biostatistics and Epidemiology, University of Massachusetts Amherst, Amherst, MA USA; 298 Department of Biostatistics, Epidemiology and Informatics, University of Pennsylvania Perelman School of Medicine, Philadelphia, PA USA; 299 TUM School of Medicine and Health, Technical University of Munich and Klinikum Rechts der Isar, Munich, Germany; 300 Present Address: Regeneron Genetics Center, Tarrytown, NY USA; 301 Present Address: Genentech, South San Francisco, CA USA

**Keywords:** Diabetes, Genome-wide association studies, Computational biology and bioinformatics, Genetic markers

## Abstract

Type 2 diabetes (T2D) is a heterogeneous disease that develops through diverse pathophysiological processes^1,2^ and molecular mechanisms that are often specific to cell type^3,4^. Here, to characterize the genetic contribution to these processes across ancestry groups, we aggregate genome-wide association study data from 2,535,601 individuals (39.7% not of European ancestry), including 428,452 cases of T2D. We identify 1,289 independent association signals at genome-wide significance (*P* < 5 × 10^−8^) that map to 611 loci, of which 145 loci are, to our knowledge, previously unreported. We define eight non-overlapping clusters of T2D signals that are characterized by distinct profiles of cardiometabolic trait associations. These clusters are differentially enriched for cell-type-specific regions of open chromatin, including pancreatic islets, adipocytes, endothelial cells and enteroendocrine cells. We build cluster-specific partitioned polygenic scores^5^ in a further 279,552 individuals of diverse ancestry, including 30,288 cases of T2D, and test their association with T2D-related vascular outcomes. Cluster-specific partitioned polygenic scores are associated with coronary artery disease, peripheral artery disease and end-stage diabetic nephropathy across ancestry groups, highlighting the importance of obesity-related processes in the development of vascular outcomes. Our findings show the value of integrating multi-ancestry genome-wide association study data with single-cell epigenomics to disentangle the aetiological heterogeneity that drives the development and progression of T2D. This might offer a route to optimize global access to genetically informed diabetes care.

## Abstract (web summary)

A meta-analysis of genome-wide association studies of type 2 diabetes (T2D) identifies more than 600 T2D-associated loci; integrating physiological trait and single-cell chromatin accessibility data at these loci sheds light on heterogeneity within the T2D phenotype.

## Main

Diabetes mellitus is a huge public-health burden, with an estimated prevalence of 537 million adults worldwide in 2021, of whom more than 90% are affected by T2D^6^. The biological processes through which T2D develops are diverse and include impaired insulin secretion and insulin resistance. This aetiological heterogeneity leads to substantial variability in patient phenotypes, including age of disease onset, manifestation of disease complications and response to management strategies^1,2^. Although environment and lifestyle are well-established risk factors for T2D, heritability has been estimated to be 69% amongst individuals of 35–60 years of age^7^. Previous genome-wide association studies (GWASs) of T2D have identified more than 500 risk loci^8,9^, which showed variable patterns of association with clinical features mediated by effector genes acting through distinct molecular mechanisms that are often cell-type specific^3,4^. Through the newly established Type 2 Diabetes Global Genomics Initiative, we present findings from a very large meta-analysis of T2D GWAS data, comprising more than 2.5 million individuals of diverse ancestry—an increase of nearly threefold in the effective sample size compared with previous efforts^8,9^. We take advantage of the power afforded by this increased sample size and combine the GWAS data with emerging single-cell functional genomics data derived from disease-relevant tissues to uncover the aetiological heterogeneity of T2D. Furthermore, we construct partitioned polygenic scores (PSs)^5^ across multiple ancestry groups, and assess their association with T2D-related macrovascular outcomes and progression to microvascular complications.

## Study overview

**Extended Data Fig. 1:**
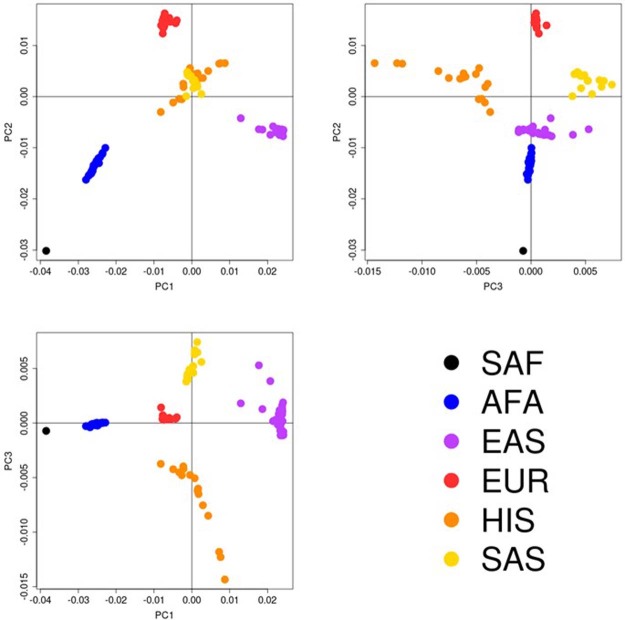
Axes of genetic variation separating GWASs of T2D across ancestry groups. We used SNVs that were reported in all studies to construct a distance matrix of mean effect allele frequency differences between each pair of GWASs. We implemented multi-dimensional scaling of the distance matrix to principal components that represent axes of genetic variation. The first three axes of genetic variation (PC1, PC2 and PC3) from multi-dimensional scaling of the Euclidean distance matrix between populations are sufficient to separate GWASs from six ancestry groups: African American (AFA), East Asian (EAS), European (EUR), Hispanic (HIS), South African (SAF), and South Asian (SAS). Variance explained by each axis: PC1 90.7%; PC2 6.5%; PC3 1.0%.

We assembled GWAS data, including 428,452 cases of T2D and 2,107,149 controls (Supplementary Fig. 1 and Supplementary Tables 1 and 2). We organized these GWASs into six subsets of genetically similar studies, which we refer to as ‘ancestry groups’ (Extended Data Fig. 1). Specifically, we considered: a European ancestry group (EUR, 60.3% of the effective sample size); an East Asian ancestry group (EAS, 19.8%); an admixed African American group with ancestry predominantly from West Africa and Europe (AFA, 10.5%); an admixed Hispanic group with ancestry predominantly from the Americas, West Africa and Europe (HIS, 5.9%); a South Asian ancestry group (SAS, 3.3%); and a South African ancestry group (SAF, 0.2%). Association analyses accounted for study-level population structure and relatedness, and adjusted for age and sex, where appropriate, and additional study-specific covariates (Supplementary Table 3 and Methods).

## Discovery of T2D loci

We aggregated association summary statistics across GWASs through multi-ancestry meta-regression, implemented in MR-MEGA (ref. ^10^), which allows for allelic effect heterogeneity that is correlated with ancestry. We included three axes of genetic variation as covariates in the meta-regression model that separated GWASs from different ancestry groups (Extended Data Fig. 1 and Methods), which resulted in lower genomic control inflation than did a fixed-effects meta-analysis (*λ*_GC_ = 1.120 and *λ*_GC_ = 1.396, respectively).

The DIAMANTE Consortium previously advocated the use of a multi-ancestry genome-wide significance threshold (*P* < 5 × 10^−9^) to define loci, which takes account of the weaker linkage disequilibrium (LD) between single-nucleotide variants (SNVs) expected after multi-ancestry meta-analysis^9^. To gain insight into true positive signals meeting conventional genome-wide significance (*P* < 5 × 10^−8^) that would be overlooked at this more stringent threshold, we considered loci reported by the DIAMANTE Consortium, which contributed 39.5% of the effective sample size of the current study. Of 39 loci with association signals meeting 5 × 10^−9^ ≤ *P* < 5 × 10^−8^ in the DIAMANTE Consortium analysis, 36 (92.3%) attained multi-ancestry genome-wide significance with the larger sample size available to us in the current study (Supplementary Text). We therefore focused our downstream analyses on SNVs that met the conventional genome-wide significance threshold.

We identified a total of 1,289 distinct T2D association signals (*P* < 5 × 10^−8^) that were represented by independent (*r*^2^ < 0.05) index SNVs (Supplementary Fig. 2, Supplementary Table 4 and Methods). The 1,289 association signals mapped to 611 loci, of which 145 (23.7%) loci have not to our knowledge been previously reported in GWASs of T2D. At association signals that mapped to loci not previously reported for T2D, index SNVs were predominantly common (minor allele frequency (MAF) higher than 5% in at least one ancestry group) with odds ratios (ORs) lower than 1.05 (Supplementary Fig. 3).

## Mechanistic clusters of T2D index SNVs

**Fig. 1:**
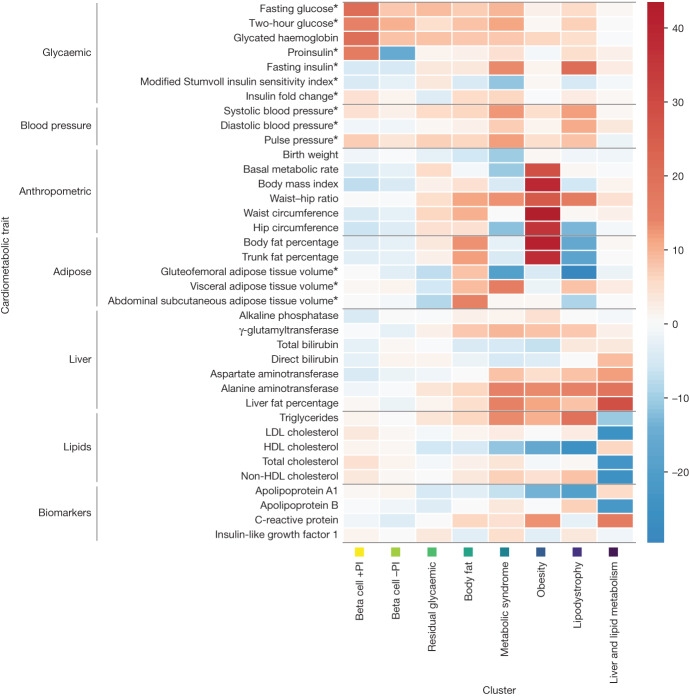
Heat map of associations of 37 cardiometabolic phenotypes with 8 mechanistic clusters of index SNVs for T2D association signals. Each column corresponds to a cluster. Each row corresponds to a cardiometabolic phenotype. The ‘temperature’ of each cell represents the *z*-score (aligned to the T2D risk allele) of association of the phenotype with index SNVs assigned to the cluster. *Phenotype is adjusted for body mass index.

**Extended Data Fig. 2:**
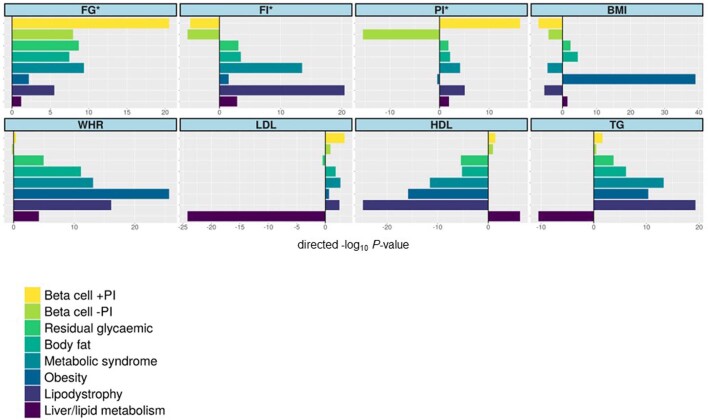
Cluster-specific associations of index SNVs with defining cardiometabolic phenotypes. Each bar presents the −log_10_ *P* value for association, with effect direction aligned to the T2D risk allele. FG: fasting glucose. FI: fasting insulin. PI: proinsulin. BMI: body mass index. WHR: waist–hip ratio. LDL: low-density lipoprotein cholesterol. HDL: high-density lipoprotein cholesterol. TG: triglycerides. *Trait adjusted for BMI.

To understand the genetic contribution to phenotypic heterogeneity in T2D, we classified the 1,289 index SNVs according to their profile of associations (aligned to the T2D risk allele) with 37 cardiometabolic phenotypes. These included glycaemic traits, anthropometric measures, body fat and adipose tissue volume, blood pressure, levels of circulating plasma lipids, and biomarkers of liver function and lipid metabolism^11–19^ (Supplementary Table 5). We applied an unsupervised ‘hard clustering’ approach with imputation of missing phenotype associations, which identified eight non-overlapping but exhaustive subsets of index SNVs with similar cardiometabolic profiles (Fig. 1, Table 1, Extended Data Fig. 2, Supplementary Fig. 4, Supplementary Tables 6 and 7 and Methods).

**Table 1:** Cardiometabolic profile, example loci and physiological effect of index SNVs at T2D association signals allocated to eight mechanistic clusters

Mechanistic cluster	Cardiometabolic profile	Number of T2D associations	Example loci	Physiological effect	
				Insulin secretion	Insulin sensitivity
Beta cell +PI	+FG*, +2hG*, +HbA1c, +PI*	91	*TCF7L2*, *KCNQ1*, *CDKAL1*, *CDKN2A–CDKN2B*, *SLC30A8*	−	+
Beta cell −PI	+FG*, +2hG*, +HbA1c, −PI*	89	*CDC123–CAMK1D*, *HNF1B*, *KCNJ11–ABCC8*, *HNF4A*, *HNF1A*	−	+
Residual glycaemic	+FG*, +HbA1c	389	*GCC1–PAX4–LEP*, *ANKRD55*, *GCKR*, *UBE2E2*	−	−
Body fat	+Body fat, +ASAT*	273	*ZMIZ1*, *HMGA2*, *CTBP1*	+	−
Metabolic syndrome	+FG*, +FI*, +WHR, +VAT*, −GFAT*, +TG, −HDL, +BP	166	*IGF2BP2*, *CCND2*, *HHEX–IDE*, *JAZF1*, *GPSM1*	+	−
Obesity	+BMI, +WHR, +body fat, +BMR, +TG, −HDL	233	*FTO*, *MC4R*, *MACF1*, *TMEM18*	+	−
Lipodystrophy	+FI*, +WHR, −body fat, −GFAT*, +TG, −HDL, +BP	45	*IRS1*, *GRB14–COBLL1*, *PPARG*	+	−
Liver and lipid metabolism	−LDL, −TC, +liver fat, +liver biomarkers	3	*TOMM40–APOE–GIPR*, *TM6SF2*, *PNPLA3*	−	−

+/−: T2D risk alleles associated with increased or decreased phenotype values.

ASAT, abdominal subcutaneous adipose tissue volume; BMI, body mass index; BMR, basal metabolic rate; BP, blood pressure; FG, fasting glucose; FI, fasting insulin; GFAT, gluteofemoral adipose tissue volume; HbA1c, glycated haemoglobin; HDL, high-density lipoprotein cholesterol; LDL, low-density lipoprotein cholesterol; PI, proinsulin; TC, total cholesterol; TG, triglycerides; VAT, visceral adipose tissue volume; WHR, waist–hip ratio; 2hG, two-hour glucose.

*Adjusted for BMI.

We observed that the cardiometabolic features and loci of five of our identified clusters overlapped with those reported in previous efforts^3,4,20,21^, representing beta-cell dysfunction with a positive or negative association with proinsulin (PI), and insulin resistance mediated through obesity, lipodystrophy, and liver and lipid metabolism (Supplementary Table 8). T2D risk alleles at index SNVs in the two beta-cell-dysfunction clusters are associated with increased fasting glucose, two-hour glucose and glycated haemoglobin, and with decreased fasting insulin. Index SNVs in both clusters are also associated with PI, but with opposite directions of effect for the T2D risk allele. The clusters reflecting mechanisms of insulin resistance mediated through obesity, lipodystrophy, and liver and lipid metabolism include index SNVs that are associated with anthropometric measures and levels of circulating plasma lipids. T2D risk alleles at index SNVs in the obesity cluster are associated with increased body mass index (BMI), waist–hip ratio (WHR), body fat percentage and basal metabolic rate, and with decreased high-density lipoprotein (HDL) cholesterol. The lipodystrophy cluster comprises index SNVs for which T2D risk alleles are associated with increased fasting insulin, WHR, blood pressure and triglycerides, and with decreased body fat percentage, gluteofemoral adipose tissue (GFAT) volume and HDL cholesterol. T2D risk alleles at index SNVs assigned to the liver and lipid metabolism cluster are associated with increased liver fat and liver-related biomarkers, and with decreased low-density lipoprotein (LDL) cholesterol and total cholesterol.

**Extended Data Fig. 3:**
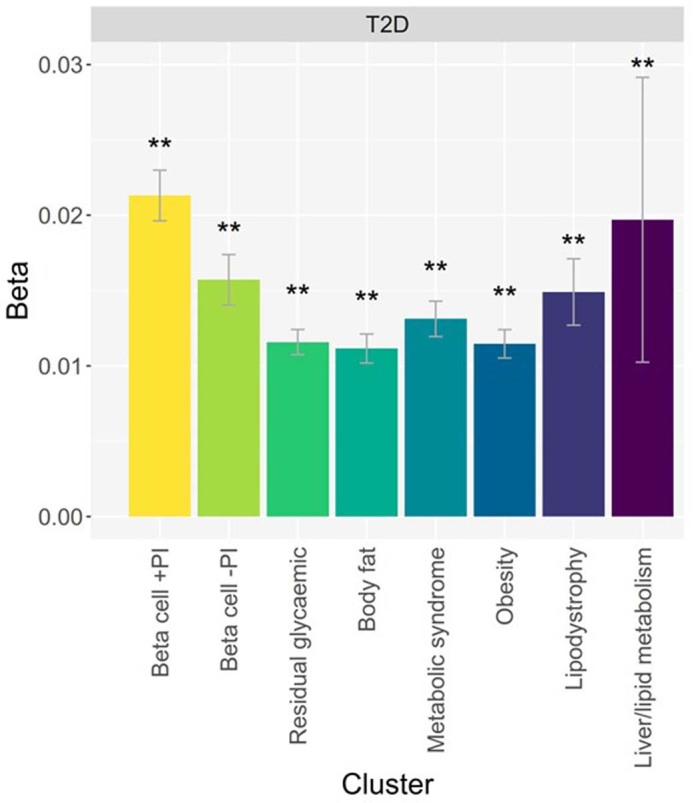
Cluster-specific associations of index SNVs with T2D. The height of each bar corresponds to the log-odds ratio (beta), and the grey bar shows the 95% confidence interval. *P < 0.05, nominal association. **P < 0.0063, Bonferroni correction for eight clusters. Exact *P* values are presented in Supplementary Table 9.

By increasing the number of index SNVs in the clustering by nearly fourfold relative to previous efforts, we provide a more granular view of the biological processes through which T2D associations affect disease, and highlight three previously unreported clusters of signals with cardiometabolic profiles that are representative of metabolic syndrome, body fat and residual glycaemic effects. We observed significantly weaker allelic effects on T2D in these three clusters than in those previously reported (mean OR of 1.028 versus 1.033, *P* = 2.2 × 10^−7^), but there was no noticeable difference in disparity around the centroid between clusters (Extended Data Fig. 3, Supplementary Table 9 and Supplementary Fig. 5). T2D risk alleles at index SNVs assigned to the metabolic syndrome cluster are associated with increased fasting glucose, WHR, triglycerides and blood pressure, and with decreased HDL cholesterol, which together are used to define metabolic syndrome. T2D risk alleles in this cluster are also associated with increased fasting insulin, with accumulations of unhealthy fat depots (increased visceral adipose tissue (VAT) volume and liver fat) and with decreased GFAT volume. Previous investigations have shown that individuals with metabolic syndrome are at increased risk of T2D^22^, although Mendelian randomization studies indicate that a causal effect is driven by increased waist circumference and increased fasting glucose^23^. T2D risk alleles at index SNVs assigned to the body fat cluster are associated with increased abdominal subcutaneous adipose tissue volume, VAT volume and body fat percentage. Although the body fat cluster profile of associations with cardiometabolic phenotypes shares these features in common with obesity-mediated insulin resistance, index SNVs in the body fat cluster are not strongly associated with BMI, lipid levels or basal metabolic rate. Previous investigations have highlighted that body fat percentage is predictive of abnormal blood glucose in individuals with a healthy BMI^24^. Finally, T2D risk alleles at index SNVs assigned to the residual glycaemic cluster are most strongly associated with increased fasting glucose and glycated haemoglobin, but, unlike the two beta-cell-dysfunction clusters, are not associated with PI or decreased fasting insulin.

**Extended Data Fig. 4:**
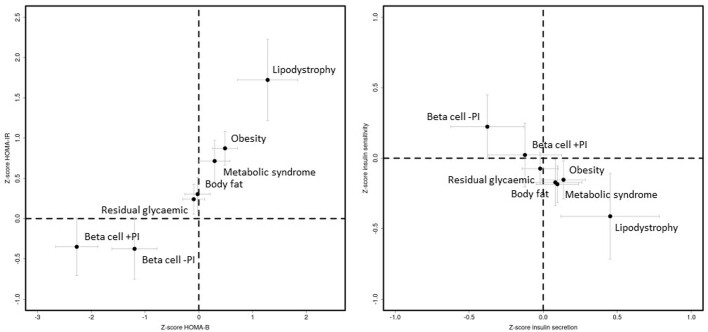
Cluster-specific associations of T2D risk alleles at index SNVs with insulin-related endophenotypes. Measures of insulin secretion and insulin sensitivity were derived from hyperinsulinaemic-euglycaemic clamp assessments and oral glucose tolerance tests in up to 1,316 Mexican American participants without diabetes. Homeostatic model assessment measures of beta-cell function (HOMA-B) and insulin resistance (HOMA-IR) were obtained from 36,466 non-diabetic individuals of European ancestry. Each point corresponds to the cluster-specific mean *z*-score for each trait, and grey bars represent 95% confidence intervals. The liver and lipid metabolism cluster has been removed for ease of presentation.

**Extended Data Fig. 5:**
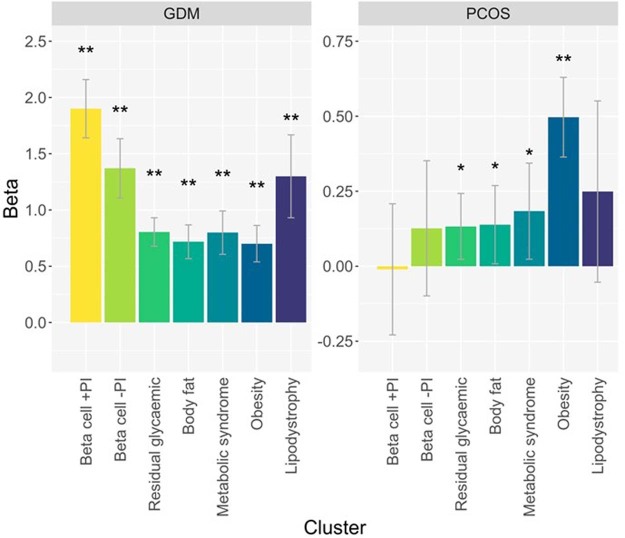
Cluster-specific associations of T2D risk alleles at index SNVs with insulin-resistance-related disorders. Association with gestational diabetes mellitus (GDM) was assessed in 5,485 cases and 347,856 female controls of diverse ancestry. Association with polycystic ovary syndrome (PCOS) was assessed in 10,074 cases and 103,164 female controls of European ancestry. The height of each bar corresponds to the mean *z*-score, and the grey bar shows the 95% confidence interval. The liver and lipid metabolism cluster has been removed for ease of presentation. *P < 0.05, nominal association. **P < 0.0063, Bonferroni correction for eight clusters. Exact *P* values are presented in Supplementary Table 12.

Clustering provides a framework to better understand the diverse physiological processes through which T2D develops and the shared biological pathways that drive genetic correlations with other insulin-resistance-related disorders, including gestational diabetes mellitus (GDM) and polycystic ovary syndrome (PCOS). T2D risk alleles at index SNVs showed a gradient of effects on insulin-related endophenotypes across clusters (Supplementary Text, Extended Data Fig. 4 and Supplementary Tables 10 and 11), representing a cline from insulin production and processing in the two beta-cell-dysfunction clusters through to insulin resistance that was most extreme in the lipodystrophy cluster. Index SNVs in the beta cell +PI cluster showed the strongest associations with GDM, whereas those in the obesity cluster were most strongly associated with PCOS (Supplementary Text, Extended Data Fig. 5 and Supplementary Table 12).

## Regulatory processes underlying clusters

**Fig. 2:**
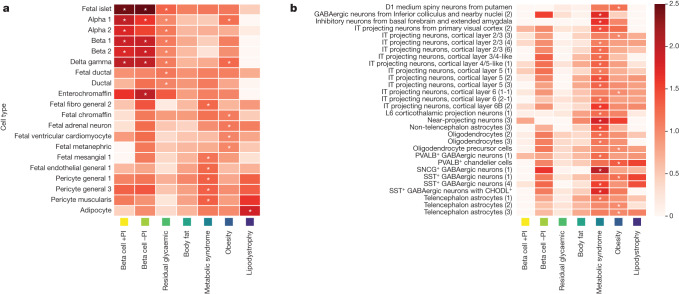
Heat map of cluster-specific enrichments of T2D associations for cell-type-specific regions of open chromatin derived from single-cell ATAC-seq peaks in adult and fetal tissue. **a**, Cell types (222 types) from 30 human adult tissues and 15 human fetal tissues. **b**, Cell types (106 types) from the human brain. In each panel, columns represent mechanistic clusters. Each row represents a cell type that was significantly enriched (Bonferroni correction for the number of cell types) for T2D associations in at least one cluster (indicated by an asterisk). The ‘temperature’ of each cell defines the magnitude of the log fold enrichment. The liver and lipid metabolism cluster is not presented because it includes only three T2D association signals and the model parameter estimates were unstable.

To gain insight into tissue-specific regulatory processes underpinning mechanistic clusters, we integrated T2D association signals with assay for transposase-accessible chromatin using sequencing (ATAC-seq) peaks from single-cell atlases of chromatin accessibility (CATLAS and DESCARTES) for 222 cell types derived from 30 human adult and 15 human fetal tissues^25,26^ and an additional 106 cell types from the human brain^27^ (Fig. 2, Supplementary Tables 13 and 14 and Methods).

We observed significant enrichment for regions of open chromatin in fetal islets and adult neuroendocrine cells in pancreatic islets (alpha, beta, gamma and delta) in the beta cell +PI, beta cell −PI and residual glycaemic clusters. In addition, the residual glycaemic cluster was enriched in fetal and adult pancreatic ductal cells, whereas the beta cell −PI cluster was enriched in adult enterochromaffin cells—a type of enteroendocrine cell that has an essential role in regulating intestinal motility and secretion in the gastrointestinal tract^28^. Enterochromaffin cells are a major target for glucagon-like peptide 1 (GLP-1) and highly express the GLP-1 receptor, agonists of which are widely used as medications for T2D^29^ (Supplementary Text).

The obesity cluster was also significantly enriched for regions of open chromatin in adult pancreatic islets, although not as strongly as were the beta-cell-dysfunction clusters. Enrichment was observed only for alpha, gamma and delta cells, suggesting that there are alternative pathways through which islets affect the development of T2D, other than through the secretion of insulin from beta cells. The obesity cluster was further enriched in fetal adrenal gland cells (chromaffin cells and adrenal neurons), fetal heart cells (ventricular cardiomyocytes) and fetal kidney cells (metanephric cells). Previous studies have reported an enrichment of BMI loci or heritability for epigenomic annotations in pancreatic islets and adrenal gland^30,31^, consistent with our findings. In the human brain, the obesity cluster was significantly enriched for regions of open chromatin in cell types including intratelencephalic (IT) projecting neurons, somatostatin-positive (SST^+^) GABAergic inhibitory neurons and D1 medium spiny neurons. SST^+^ GABAergic neurons exist in the hypothalamus and regulate food intake^32^. D1 medium spiny neurons are a type of GABAergic neuron in the human striatum that expresses D1-type dopamine receptors; these neurons have been implicated in food motivation and the development of diet-induced obesity in mice^33^.

The remaining four clusters (lipodystrophy; metabolic syndrome; body fat; and liver and lipid metabolism) were not significantly enriched for regions of open chromatin in pancreatic islets. The lipodystrophy cluster was enriched only in adult adipocytes, which confirms previous reports in bulk adipose tissue^4,20^. Consistent with these results, association signals for WHR, triglycerides and HDL cholesterol, which are strongly affected by index SNVs in the lipodystrophy cluster, have been shown to be enriched in candidate *cis*-regulatory elements in adipocytes^26^. The metabolic syndrome cluster was enriched in cells that reside in the walls of blood vessels (adult pericytes and fetal endothelial cells), fetal kidney cells (mesangial cells) and fetal fibroblasts. Association signals for systolic and diastolic blood pressure, a key component of metabolic syndrome, have been shown to be enriched in candidate *cis*-regulatory elements in these cell types^26^. Endothelial dysfunction is not only a consequence of insulin resistance, but also impairs insulin signalling to further reduce insulin sensitivity, thereby providing a pathophysiological mechanism that links the metabolic and cardiovascular components of metabolic syndrome^34^. In human brain, the metabolic syndrome cluster was significantly enriched for regions of open chromatin in cell types including IT projecting neurons and SST^+^ GABAergic inhibitory neurons. IT projecting neurons are a type of glutamatergic excitatory pyramidal neuron in the cerebral cortex, and metabolic syndrome was previously associated with pyramidal neurons and GABAergic neurons in cell-type specificity analyses in a GWAS that examined genetic factors in metabolic syndrome^35^. We observed no significant enrichments in the body fat cluster or in the liver and lipid metabolism cluster.

## Ancestry-correlated heterogeneity

Previous multi-ancestry GWASs have shown widespread heterogeneity in allelic effects at T2D association signals across ancestry groups^9,36^. We took advantage of the meta-regression model to partition heterogeneity into an ancestry-correlated component explained by three axes of genetic variation, and a residual component reflecting differences in environmental exposures (that are not correlated with ancestry) and/or study design (Supplementary Table 15). We observed 127 (9.9%) independent T2D association signals with significant evidence for ancestry-correlated heterogeneity (*P*_HET_ < 3.9 × 10^−5^, Bonferroni correction for 1,289 signals). We would expect less than one signal to meet this threshold of significance, highlighting that ancestry-correlated heterogeneity is strongly enriched at T2D associations (one-sided binomial test *P* < 2.2 × 10^−16^). By contrast, we observed significant evidence of residual heterogeneity at only four (0.3%) association signals (one-sided binomial test *P* = 0.031). These results therefore suggest that differences in allelic effects at index SNVs are more strongly correlated with genetic ancestry than other factors that vary between GWASs.

**Extended Data Fig. 6:**
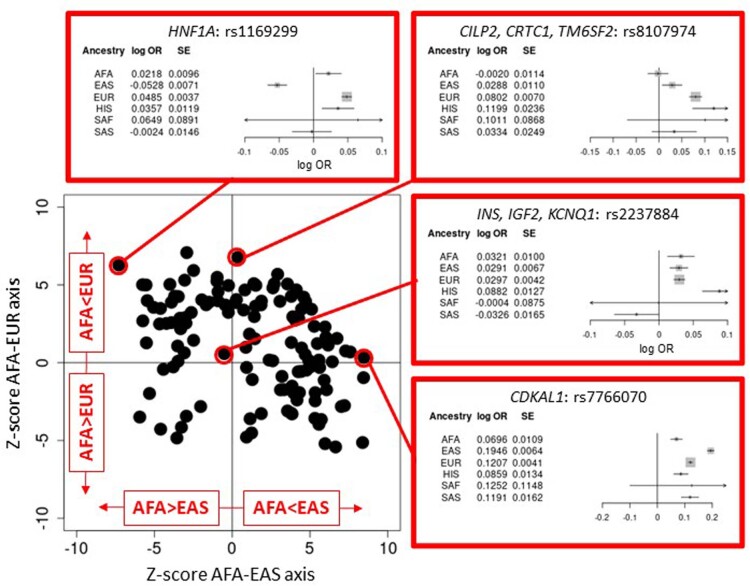
Ancestry-correlated heterogeneity is driven by differences in allelic effect sizes between AFA, EAS and EUR ancestry groups. In the scatter plot, index SNVs with significant evidence (*P*_HET_ < 3.9 × 10^−5^, Bonferroni correction for 1,289 signals) for ancestry-correlated heterogeneity are plotted according to their association (*z*-score) with the first two axes of genetic variation. The first axis represents differences in allelic effect sizes between AFA/EUR GWASs and EAS GWASs (AFA–EAS axis), whilst the second axis represents differences in effect size between AFA/EAS GWASs and EUR GWASs (AFA–EUR axis). The forest plots present examples of ancestry-correlated heterogeneity at index SNVs. In each forest plot, the allelic log-odds ratio (OR) from each ancestry group-specific fixed-effects meta-analysis is given by the black tick mark, the 95% confidence interval is given by the horizontal line, and the weight (inverse-variance) of each ancestry group by the grey box. AFA: African American ancestry group. EAS: East Asian ancestry group. EUR: European ancestry group. HIS: Hispanic ancestry group. SAF: South African ancestry group. SAS: South Asian ancestry group.

We next sought to better understand the impact of genetic diversity on differences in allelic effects between GWASs at the 127 association signals with significant evidence of ancestry-correlated heterogeneity (Methods). For 118 (92.9%) signals, allelic effect sizes were most strongly associated with the first two axes of genetic variation, which reflect differences between AFA/EUR and EAS GWASs (AFA–EAS axis), and between AFA/EAS and EUR GWASs (AFA–EUR axis), respectively (Supplementary Text, Extended Data Figs. 1 and 6 and Supplementary Table 16).

**Extended Data Fig. 7:**
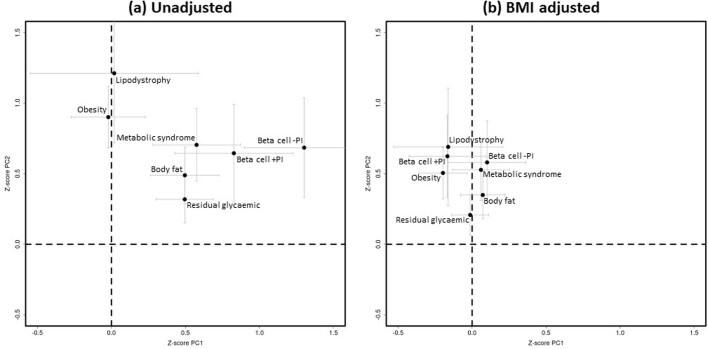
Cluster-specific associations of index SNVs with the first two axes of genetic variation in T2D cases and controls. **a**, Unadjusted for BMI. **b**, Adjusted for study-level mean BMI. Each point corresponds to a cluster, plotted according to the mean *z*-score for association with the first two axes of genetic variation (PC1 and PC2) on the *x* axis and *y* axis, respectively. Grey bars correspond to 95% confidence intervals. The liver and lipid metabolism cluster has been removed for ease of presentation.

We observed significant differences in mean *z*-scores for association between clusters for both the AFA–EAS axis (*P* = 4.1 × 10^−6^) and the AFA–EUR axis (*P* = 1.5 × 10^−6^). Index SNVs in the two beta-cell-dysfunction clusters were most positively associated with the AFR–EAS axis, indicating allelic effects on T2D that were greater in EAS GWASs than in AFA and EUR GWASs (Extended Data Fig. 7 and Supplementary Table 17). By contrast, index SNVs in the lipodystrophy and obesity clusters were most positively associated with the AFA–EUR axis, indicating allelic effects on T2D that were greater in EUR GWASs than in EAS and AFA GWASs. These results indicate that ancestry-correlated heterogeneity varies between mechanistic clusters, with allelic effects greatest for EAS GWASs at association signals assigned to clusters acting through beta-cell dysfunction and greatest for EUR GWASs at those assigned to clusters operating through insulin resistance.

Ancestry-correlated heterogeneity in allelic effects between GWASs is not driven by differences in allele frequency between ancestry groups, but can occur because of interaction between index SNVs and environmental and lifestyle factors, if not accounted for in the association analysis^37^. We observed substantial variation in the distribution of study-level mean BMI in T2D cases and controls across ancestry groups (Supplementary Fig. 6). Such variation could affect ancestry-correlated heterogeneity because, when cases and controls are selected from the extremes of the BMI distribution, the magnitude of allelic effect estimates at T2D signals acting through beta-cell dysfunction can be inflated^38^. We therefore extended the MR-MEGA meta-regression model to allow for allelic effect heterogeneity at index SNVs due to mean BMI in T2D cases and controls, in addition to axes of genetic variation (Methods).

After adjustment for study-level mean BMI in cases of T2D and in controls, only 24 association signals retained significant evidence of ancestry-correlated heterogeneity (*P* < 3.9 × 10^−5^), compared with 127 signals without adjustment (Supplementary Text and Supplementary Table 18). After adjustment for BMI, significant differences in mean *z*-scores for association between clusters for the AFA–EUR axis were maintained (*P* = 3.2 × 10^−5^ versus *P* = 1.5 × 10^−6^ without adjustment), whereas those for the AFA–EAS axis were not (*P* = 0.18 versus *P* = 4.1 × 10^−6^ without adjustment). Furthermore, after adjustment for BMI, the two beta-cell-dysfunction clusters were no longer strongly positively associated with the AFA–EAS axis (Extended Data Fig. 7 and Supplementary Table 19). Together, these results suggest that heterogeneity in allelic effects between EAS GWASs and EUR/AFA GWASs, which occur most often at association signals assigned to the beta-cell-dysfunction clusters, can be mostly accounted for by differences in the distributions of mean BMI in T2D cases and in controls between these ancestry groups.

## Associations of partitioned PS with outcomes

**Fig. 3:**
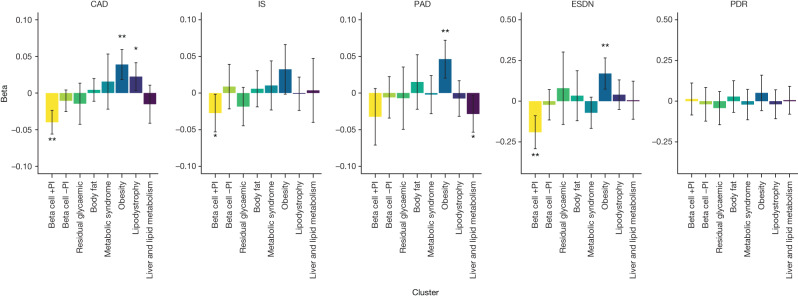
Associations of cluster-specific components of the partitioned PS with five T2D-related vascular outcomes in up to 279,552 individuals from multiple ancestry groups. Summaries of the associations of each cluster-specific component of the partitioned PS with CAD, ischaemic stroke (IS), peripheral artery disease (PAD), ESDN and proliferative diabetic retinopathy (PDR). The height of each bar corresponds to the log-odds ratio (beta) per standard deviation of the PS, and the grey bar shows the 95% confidence interval. Analyses of T2D-related macrovascular complications (CAD, PAD and IS) were undertaken in all individuals, with adjustment for T2D status. Analyses of microvascular complications were undertaken in individuals with T2D only. *P < 0.05, nominal association; **P < 0.0063, Bonferroni correction for eight clusters. Exact *P* values are provided in Supplementary Table 21.

The major complications in individuals with T2D are macrovascular outcomes including coronary artery disease (CAD), ischaemic stroke and peripheral artery disease, and microvascular outcomes, including end-stage diabetic nephropathy (ESDN) and proliferative diabetic retinopathy. We tested for association of a cluster-specific partitioned PS with these vascular outcomes in up to 279,552 individuals (including 30,288 cases of T2D) across five ancestry groups (AFA, EAS, EUR, HIS and SAS) from the All of Us Research Program, Biobank Japan and the Genes & Health study (Methods). These individuals were not included in the multi-ancestry meta-analysis, thus avoiding potential inflated type I error rates owing to overlap between the discovery and the testing datasets. To maximize sample size, we tested macrovascular outcomes in all individuals, adjusted for T2D status, and microvascular complications only in individuals with T2D (Methods and Supplementary Table 20). To assess the additional information afforded by the partitioned PS over an overall T2D PS, agnostic to cluster membership, we tested for association of each cluster-specific component of the partitioned PS after adjustment for the overall PS. Figure 3 provides an overview of the associations of each cluster-specific component of the partitioned PS with the five vascular outcomes across ancestry groups.

**Extended Data Fig. 8:**
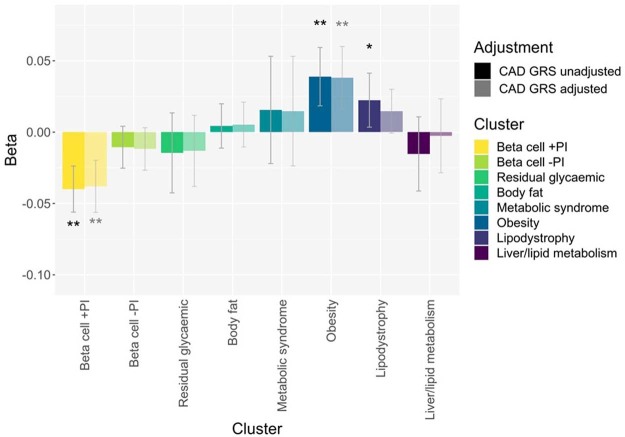
Associations of cluster-specific components of the partitioned PS with CAD in up to 279,552 individuals across diverse ancestry groups. The panel summarizes the associations of each cluster-specific component of the partitioned PS with CAD, with and without adjustment for a previously published multi-ancestry CAD PS. The height of each bar corresponds to the log-OR (beta) per standard deviation of the PS, and the grey bar shows the 95% confidence interval. Analyses were undertaken in all individuals, with adjustment for T2D status. *P < 0.05, nominal association. **P < 0.0063, Bonferroni correction for eight clusters. Exact *P* values are presented in Supplementary Tables 21 and 22.

We observed a significant association (*P* < 0.0063, Bonferroni correction for eight clusters) of two components of the partitioned PS with CAD: a negative association with the beta cell +PI cluster (OR = 0.96 per standard deviation of the PS, *P* = 1.3 × 10^−6^) and a positive association with the obesity cluster (OR = 1.04, *P* = 0.00019). There was no evidence of heterogeneity in the effects of these two clusters on CAD across ancestry groups (Supplementary Fig. 7 and Supplementary Table 21). Notably, after adjustment for a CAD PS derived from a previously published multi-ancestry meta-analysis of CAD GWASs^39^, the positive CAD association with both components of the partitioned PS remained significant (Extended Data Fig. 8 and Supplementary Table 22): beta cell +PI cluster (OR = 0.96, *P* = 4.4 × 10^−5^) and obesity cluster (OR = 1.04, *P* = 0.00065). We also observed a significant positive association of the obesity cluster from the partitioned PS with peripheral artery disease (OR = 1.05, *P* = 0.00045), with no evidence of heterogeneity in effects across ancestry groups (Supplementary Fig. 8 and Supplementary Table 21). Across all three macrovascular outcomes, there was a general trend of negative association with the beta cell +PI cluster and positive association with the obesity cluster, although no cluster-specific components of the partitioned PS attained significance for ischaemic stroke (Supplementary Fig. 9 and Supplementary Table 21). There was no strong association of the overall T2D PS with CAD (*P* = 0.17), ischaemic stroke (*P* = 0.022) or peripheral artery disease (*P* = 0.77) after meta-analysis across ancestry groups. Together, these results highlight the advantages of the partitioned PS over an overall T2D PS for detecting associations with macrovascular outcomes, and provide insight into the biological processes that lead to their development.

We observed significant associations of two components of the partitioned PS with ESDN: a negative association with the beta cell +PI cluster (OR = 0.83, *P* = 0.00024) and a positive association with the obesity cluster (OR = 1.19, *P* = 0.00050). There was no evidence of heterogeneity in the effects of these two clusters across ancestry groups, (Supplementary Fig. 10 and Supplementary Table 21), and the overall PS was not strongly associated with ESDN (*P* = 0.048). By contrast, none of the cluster-specific components of the partitioned PS were associated with proliferative diabetic retinopathy. However, there was a strong positive association of the overall PS with this microvascular outcome (OR = 1.32, *P* = 1.1 × 10^−9^), with no evidence of heterogeneity in effects across ancestry groups (Supplementary Fig. 11 and Supplementary Table 21). Together, these results suggest that ESDN is associated with obesity and beta-cell dysfunction with opposite directions of effect, and confirm previous reports that proliferative diabetic retinopathy is driven by hyperglycaemia^40^ and therefore strongly associated with the overall burden of T2D risk variants.

**Extended Data Fig. 9:**
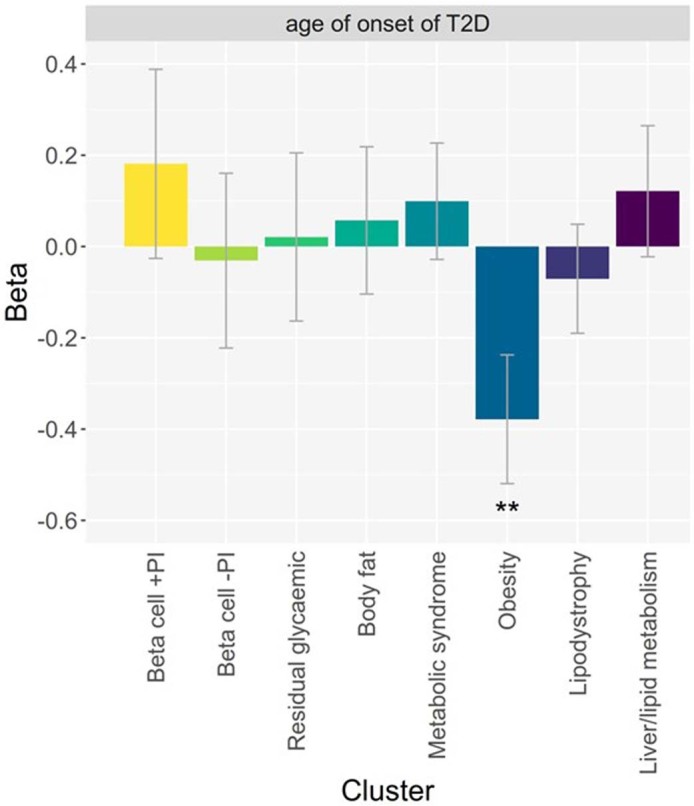
Associations of cluster-specific components of the partitioned PS with T2D age of onset in up to 30,288 individuals across diverse ancestry groups. The panel summarizes the associations of each cluster-specific component of the partitioned PS with age of onset. The height of each bar corresponds to years (beta) per standard deviation of the PS, and the grey bar shows the 95% confidence interval. A negative effect corresponds to earlier age of onset. *P < 0.05, nominal association. **P < 0.0063, Bonferroni correction for eight clusters. Exact *P* values are presented in Supplementary Table 23.

Finally, we tested for associations of the cluster-specific components of the partitioned PS and the overall T2D PS with age of onset of T2D (Extended Data Fig. 9 and Methods). The overall PS was strongly associated with an earlier age of onset (1.15 years per standard deviation of the PS, *P* = 5.1 × 10^−8^), although the effects were highly heterogeneous across ancestry groups (Supplementary Fig. 12 and Supplementary Table 23). However, even after adjustment for the overall PS, the obesity cluster was significantly associated with an earlier age of onset (0.38 years, *P* = 1.4 × 10^−7^), with no evidence of heterogeneity across ancestry groups. These findings highlight the importance of obesity-related processes for the onset of T2D, in addition to the development of vascular complications.

## Associations with vascular outcomes in clinical trials

**Extended Data Fig. 10:**
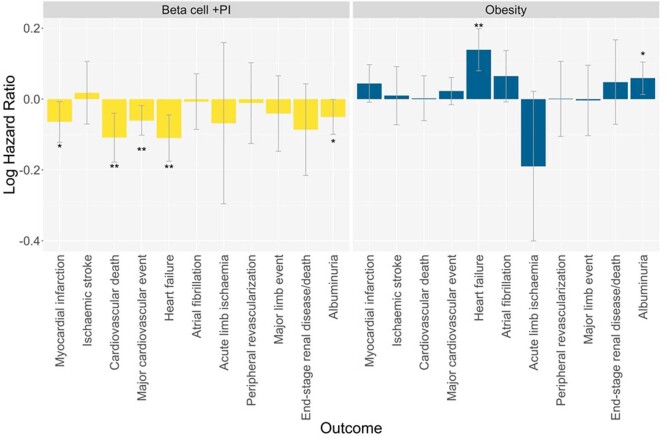
Associations of the beta cell +PI and obesity cluster-specific components of the partitioned PS with vascular outcomes in up to 29,827 EUR individuals with T2D from six clinical trials from the TIMI Study Group. Major cardiovascular event is defined as myocardial infarction, ischaemic stroke, or cardiovascular death. Major limb event is defined as acute limb ischaemia or peripheral revascularization. The height of each bar corresponds to the log-hazard ratio per standard deviation of the PS, and the grey bar shows the 95% confidence interval. *P < 0.05, nominal association. **P < 0.0063, Bonferroni correction for eight clusters. Exact *P* values are presented in Supplementary Table 24.

To gain insight into the associations of the obesity and beta cell +PI clusters with a broader range of vascular outcomes, we assessed the performance of the partitioned PS (after adjustment for the overall PS) in prospective GWASs in up to 29,827 EUR individuals with T2D from six clinical trials from the Thrombolysis in Myocardial Infarction (TIMI) Study Group (Methods and Supplementary Table 24). We observed the strongest associations of cluster-specific components of the partitioned PS with risk of hospitalization for heart failure: positive with the obesity cluster (hazard ratio (HR) = 1.15 per standard deviation of the PS, *P* = 4.8 × 10^−6^) and negative with the beta cell +PI cluster (HR = 0.90, *P* = 0.00092). Amongst macrovascular outcomes, the beta cell +PI cluster was also negatively associated with cardiovascular death (HR = 0.90, *P* = 0.0020), major cardiovascular events (HR = 0.94, *P* = 0.0050) and myocardial infarction (HR = 0.94, *P* = 0.027). For microvascular outcomes, the two clusters showed associations with opposite directions of effect for albuminuria: obesity cluster (HR = 1.06, *P* = 0.012) and beta cell +PI cluster (HR = 0.95, *P* = 0.047). Across all outcomes, there was a general trend of positive association with the obesity cluster and negative association with the beta cell +PI cluster (Extended Data Fig. 10), consistent with the associations observed from our analyses of retrospective GWASs across ancestry groups.

## Discussion

To better understand the aetiological heterogeneity of T2D across diverse populations, we assembled a large collection of T2D GWASs for six ancestry groups through the Type 2 Diabetes Global Genomics Initiative. By increasing the effective sample size by almost threefold compared with previous efforts, we identified a total of 611 loci attaining the conventional threshold of genome-wide significance (*P* < 5 × 10^−8^), 145 (23.7%) of which have not to our knowledge been previously reported. This conventional threshold is equivalent to a Bonferroni correction for the effective number of independent SNVs in EUR reference data^41^. Using empirical data from the 1000 Genomes Project, the DIAMANTE Consortium and others have advocated more stringent thresholds for multi-ancestry meta-analysis because the structure of LD is broken down across ancestry groups and the effective number of independent SNVs is increased^9,42^. In fact, our analyses suggest that loci meeting conventional genome-wide significance are unlikely to be false positive association signals, but instead are driven by index SNVs that have modest effects that require larger sample sizes to meet more stringent thresholds. We therefore recommend the use of this conventional threshold but advocate careful review of reported signals to ensure that associations are not driven by single studies or poorly imputed variants to protect against false positives.

Multi-ancestry meta-regression maximizes power to detect associations that are shared across ancestry groups by allowing for heterogeneity in allelic effects at index SNVs. MR-MEGA is not restricted to broad continental ancestry labels that can be used to reinforce the concept of fundamental genetic differences between groups^43^, but instead represents ancestry as continuous axes of genetic variation, which better reflect the continuum of human genetic diversity and demographic history^44^. Still, it is important to emphasize that our meta-analysis does not fully capture global genetic diversity, in particular underrepresented populations across Africa, South and Central America, the Middle East and Oceania. For example, 98.2% of the total effective sample size of individuals with the highest proportion of ancestry from Africa are African Americans. The ancestry of these individuals represents a cline of admixture that is predominantly from West Africa and is therefore not representative of other regions in Africa, where the level of genetic variation is equivalent to the differences observed between other continental groups^43^. Bolstering GWAS collections in these underrepresented populations remains an urgent priority for the human genetics research community and highlights the need for careful interpretation of results that does not generalize findings across ancestry groups that are sensitive to biased representation.

Within the landscape of the genetic architecture of T2D, we identified eight clusters of index SNVs with distinct profiles of associations with 37 cardiometabolic phenotypes, which defined pathophysiology-relevant groupings. The addition of previously unreported T2D signals identified through the multi-ancestry meta-analysis helped define three clusters that were not detected in previous clustering efforts^3,4,20,21^, with cardiometabolic profiles that are consistent with residual glycaemic effects, accumulations of body fat and metabolic syndrome. These previous efforts have implemented ‘soft clustering’ approaches, such as Bayesian non-negative matrix factorization, that generate weights for cluster membership for each index SNV^4^. The assignment of index SNVs to clusters is then determined given a threshold weight for cluster membership, allowing for the possibility that a T2D association signal affects disease through multiple pathophysiological pathways. However, depending on the threshold for cluster membership, some index SNVs will be unassigned. Bayesian non-negative matrix factorization also considers positive and negative associations with the same phenotype as independent variables, and most clustering methods cannot directly accommodate missing phenotype associations. To address these potential limitations, we implemented methodology that jointly conducts *k*-means clustering of index SNVs with powerful iterative multiple imputation of missing phenotype associations. In this ‘hard clustering’ approach, each index SNV is assigned to exactly one cluster. This has the potential disadvantage, therefore, that index SNVs with outlying or intermediate profiles of trait associations are ‘forced’ into a cluster that does not fit well. However, the previously unreported clusters that we identified in our hard clustering were not noticeably more disparate than the clusters reported previously, suggesting that we have not introduced substantial noise by forcing all SNVs into exactly one cluster. Ultimately, the choice of clustering approach may depend on the objectives of any downstream investigations.

Our analyses highlighted a significant excess of T2D association signals with ancestry-correlated heterogeneity, which is driven mainly by differences in allelic effects between AFA, EAS and EUR GWASs. The two beta-cell-dysfunction clusters are most strongly associated with the AFA–EAS axis, in which effects are typically larger in EAS GWASs than in those for other ancestry groups. These two clusters are also most strongly associated with reduced insulin secretion and lower insulin resistance. By contrast, the lipodystrophy and obesity clusters, which are characterized by reduced insulin sensitivity and higher insulin resistance, are most strongly associated with the AFA–EUR axis, in which effects are typically larger in EUR than in other ancestry groups. These observations are consistent with studies reporting differences in the pathogenesis of T2D between ancestry groups, whereby T2D is initiated mainly through increased insulin resistance in EUR individuals, but is characterized by reduced insulin secretion with lower insulin resistance in EAS individuals^45,46^. We have shown that most signals with ancestry-correlated heterogeneity can be explained by differences in the distribution of BMI in T2D cases and controls between ancestry groups. Furthermore, after adjustment for study-level mean BMI, we observe no difference in allelic effects between clusters along the AFA–EAS axis. This is consistent with previous studies that reported that body composition is the main determinant of variation in T2D pathogenesis between EAS and EUR individuals, because insulin sensitivity and beta-cell response are similar in the two ancestry groups after accounting for differences in BMI^45,47^.

We reveal—across multiple ancestry groups—significant associations of vascular outcomes with cluster-specific components of the partitioned PS after adjustment for the overall PS, which suggests that disease trajectories are associated with genetic burden in certain biological pathways that are consistent across diverse populations. Although the effect sizes of the cluster-specific components of the partitioned PS were small, they motivate future work to strengthen these effects through the identification of further T2D associations in larger sample sizes. Through integration with single-cell chromatin accessibility data across diverse cell types, they also enhance understanding of key biological processes driving heterogeneity in the clinical features of T2D phenotypes. For example, the obesity-cluster-specific component of the PS was positively associated with CAD and ESDN, and included index SNVs that were enriched for regions of open chromatin in fetal ventricular cardiomyocytes, fetal adrenal neuron, adult chromaffin cells in the adrenal gland and fetal metanephric cells. These findings are in line with the reported enrichments of CAD association signals for transcriptomic and epigenomic annotations in bulk tissues including the aorta and arteries, the heart and the adrenal gland^39,48,49^, and of renal function association signals in kidney-tissue-specific regulatory annotations^50^. Together, these findings provide a clear link to shared biological mechanisms that drive the development of T2D and other vascular diseases.

In conclusion, our findings show the value of integrating multi-ancestry GWASs of T2D and cardiometabolic traits with single-cell epigenomics across diverse tissues to disentangle the aetiological heterogeneity driving the development and progression of T2D across population groups. Improved understanding of the varied pathophysiological processes that link T2D to vascular outcomes could offer a route to genetically informed diabetes care and global opportunities for the clinical translation of findings from T2D GWASs.

## Methods

### Study-level analyses

Within each study, we assigned individuals to ancestry groups using self-report and genetic background (Supplementary Tables 1 and 2). Any individuals not assigned to an ancestry group were excluded as population outliers. Within each ancestry group-specific GWAS, we conducted quality control of genotype data and imputed up to reference panels from the Trans-Omics for Precision Medicine Program^51^, Haplotype Reference Consortium^52^, 1000 Genomes Project (phase 1, March 2012 release; phase 3, October 2014 release)^53,54^, or population-specific whole-genome sequencing^55–61^ (Supplementary Table 3). Studies imputed to reference panels mapped to GRCh38 (hg38) were lifted back to hg19 using the UCSC LiftOver tool (https://genome.ucsc.edu/cgi-bin/hgLiftOver). We excluded SNVs with poor imputation quality and/or minor allele count (MAC) < 5 (Supplementary Table 3).

Within each ancestry group-specific GWAS, we tested for association of each SNV with T2D through generalized linear (mixed) modelling, under an additive dosage of the minor allele, with adjustment for age and sex (where appropriate), and additional study-specific covariates (Supplementary Table 3). We used different strategies to account for population stratification and/or kinship: (i) exclude closely related individuals and adjust for principal components derived from a genetic relatedness matrix (GRM) as additional covariates; or (ii) incorporate a random effect for the GRM (Supplementary Table 3). Allelic effects and corresponding standard errors that were estimated from a linear mixed model were converted to the log-odds scale^62^. We corrected study-level association summary statistics for residual structure by the LD-score regression intercept^63^ (Supplementary Table 3) using an LD reference that we derived from ancestry-matched haplotypes from continental groups in the 1000 Genomes Project (phase 3, October 2014 release)^54^. We matched AFA GWASs to the ‘African’ continental group and HIS GWASs to the ‘American’ continental group.

### Multi-ancestry meta-analyses

We analysed autosomal bi-allelic SNVs that overlap reference panels from the 1000 Genomes Project (phase 3, October 2014 release)^54^ and the Haplotype Reference Consortium^52^. We considered SNVs with MAF > 0.5% in at least one of the five continental groups in the 1000 Genomes Project (phase 3, October 2014 release)^54^. We excluded SNVs that differed in allele frequency by more than 20% when comparing reference panels in the same subsets of haplotypes.

We used meta-regression, implemented in MR-MEGA^10^, to aggregate association summary statistics across GWASs. MR-MEGA models allelic effect heterogeneity that is correlated with genetic ancestry by including axes of genetic variation as covariates in the meta-regression model to capture diversity between GWASs. We used SNVs reported in all studies to construct a distance matrix of differences in mean effect allele frequency between each pair of GWASs. We implemented multi-dimensional scaling of the distance matrix to obtain three principal components that represent axes of genetic variation to separate GWASs across ancestry groups (Extended Data Fig. 1).

For each SNV, we aggregated inverse-variance weighted allelic effects across GWASs through linear regression, including three axes of genetic variation as covariates. We tested for: (i) association with T2D allowing for ancestry-correlated allelic effect heterogeneity between GWASs; (ii) ancestry-correlated allelic effect heterogeneity between GWASs (defined by the axes of genetic variation); and (iii) residual allelic effect heterogeneity between GWASs. MR-MEGA is a meta-regression approach, and therefore does not produce an allelic effect estimate because this is allowed to vary with the axes of genetic variation. Consequently, we also aggregated association summary statistics across GWASs through fixed-effects meta-analysis (inverse-variance weighting of allelic effects) using METAL^64^. To assess the extent of residual structure between GWASs, we calculated the genomic control inflation factor^65^ for the multi-ancestry meta-regression and the fixed-effects meta-analysis. We considered only those SNVs reported in at least five GWASs for downstream interrogation.

### Defining T2D signals and loci

We identified all SNVs attaining genome-wide significance (*P* < 5 × 10^−8^) for association with T2D from the multi-ancestry meta-regression. Clumps were formed around index variants, which were selected using a greedy algorithm in PLINK v.1.9 (ref. ^66^), after ranking SNVs by ascending *P* value. SNVs less than 5 Mb from an index SNV were assigned to the clump if *r*^2^ > 0.05 in at least one of the five continental groups from the 1000 Genomes Project (phase 3, October 2014 release)^54^. Index SNVs separated by less than 1 Mb were assigned to the same locus. Each locus was then defined as mapping 500 kb up- and downstream of index SNVs contained within it. We considered the locus to have been previously reported if it contained variants discovered in published large-scale T2D GWASs at genome-wide significance.

### Ancestry-group-specific meta-analyses

We aggregated association summary statistics across GWASs from the same ancestry group through fixed-effects meta-analysis (inverse-variance weighting of allelic effects) using METAL^64^. We estimated the mean effect allele frequency across GWASs from each ancestry group, weighted by the effective sample size of the study. We generated forest plots of association summary statistics of index SNVs across ancestry groups using the R package meta (https://cran.r-project.org/package=meta/).

### Defining clusters of T2D index SNVs with distinct cardiometabolic profiles

We considered cardiometabolic-related quantitative phenotypes that are used to define T2D status and/or are associated with risk of T2D or complications. We excluded phenotypes for which GWAS summary statistics were available only after imputation to reference panels from the International HapMap Project^67^ because they did not provide sufficient coverage of SNVs included in the multi-ancestry meta-analysis. We considered the largest available GWAS meta-analysis (ancestry-specific or multi-ancestry) that provided the following association summary statistics for each SNV: effect allele, other allele, allelic effect and corresponding standard error (Supplementary Table 5). We re-aligned the effect estimate to the T2D risk allele from the fixed-effects multi-ancestry meta-analysis, denoted *β*_*ij*_ for the *j*th index SNV and the *i*th phenotype. We then calculated a sample size corrected *z*-score, given by ${Z}_{ij}={\beta }_{ij}/\left(\sqrt{{N}_{i}}{s}_{ij}\right)$, where *s*_*ij*_ is the standard error of the effect estimate of the *j*th index SNV and the *i*th phenotype, and *N*_*i*_ is the maximum sample size reported for the *i*th phenotype. Where association summary statistics were not reported, the *z*-score was set as ‘missing’.

We conducted *k*-means clustering of index SNVs with imputation of missing *z*-scores using the R package ClustImpute (https://cran.r-project.org/package=ClustImpute). For a pre-defined number of clusters, ClustImpute replaces missing *z*-scores at random from the marginal distribution for the phenotype in the first iteration and performs *k*-means clustering. In subsequent iterations, missing *z*-scores are updated, conditional on the current cluster assignment, so that correlations between phenotypes are considered. At each iteration, penalizing weights are imposed on imputed values and successively decreased (to zero) as the missing data imputation improves. Finally, we determined the ‘optimal’ number of clusters according to the majority rule across 27 indices of cluster performance^68^, implemented in the R package NbClust (https://cran.r-project.org/package=NbClust).

We tested for association of the *i*th phenotype with index SNVs across clusters in a linear regression model, given by $E\left({Z}_{ij}\right)={\sum }_{k}{\gamma }_{ik}{C}_{jk}$, where *C*_*jk*_ is an indicator variable that takes the value 1 if the *j*th index SNV was assigned to the *k*th cluster and 0 otherwise. The strength or direction of the association of each phenotype with each cluster was then presented in a heat map, in which the ‘temperature’ was defined by the direction of the regression coefficient *γ*_*ik*_ and the corresponding −log_10_ *P* value. Regression models were fitted using the glm function in R.

We extracted cardiometabolic phenotype *z*-scores from the final imputed dataset from ClustImpute. We calculated the Euclidean distance between the *j*th SNV and *k*th cluster centroid as

$${\delta }_{jk}=\sqrt{{\sum }_{i}{\left({Z}_{ij}-{\mu }_{ik}\right)}^{2}},$$

where *Z*_*ij*_ and *μ*_*ik*_ are the *z*-score of the *j*th SNV and the location of the *k*th cluster centroid for the *i*th cardiometabolic phenotype. To assess cluster disparity, we also performed principal components analysis of cardiometabolic phenotype *z*-scores from the final imputed dataset using the R package factoextra (https://cran.r-project.org/package=factoextra).

### Cluster-specific associations of index SNVs with T2D

We tested for association of T2D with index SNVs across clusters in a linear regression model, given by $E\left({\beta }_{j}\right)={\sum }_{k}{\gamma }_{k}{C}_{jk}$, where *C*_*jk*_ is an indicator variable that takes the value 1 if the *j*th index SNV was assigned to the *k*th cluster and 0 otherwise, and weighted by the inverse of the variance of the allelic effect. We tested for heterogeneity in cluster effects on T2D by comparing the deviance of this model with that of $E\left({\beta }_{j}\right)={\gamma }_{0}$, again weighted by the inverse of the variance of the allelic effect. To compare associations between previously reported clusters and previously unreported clusters, we replaced *C*_*jk*_ with an indicator variable that takes the value 1 if the *j*th index SNV was assigned to a previously reported cluster and 0 otherwise. Regression models were fitted using the glm function in R.

### Enrichment of T2D associations for cell-type-specific regions of open chromatin within clusters

For each T2D association signal, we defined ‘null’ SNVs that mapped within 50 kb of the index SNV and were not in LD (*r*^2^ > 0.05) with the index SNV in any of the five continental groups from the 1000 Genomes Project (phase 3, October 2014 release)^54^. We defined an indicator variable, *Y*_*j*_, taking the value 1 if the *j*th SNV is an index SNV and 0 if the *j*th SNV is a null SNV. We mapped index SNVs and null SNVs to genic regions defined by the Ensembl Project (release 104)^69^, including protein-coding exons, and 3′ UTRs and 5′ UTRs. We defined indicator variables, ${G}_{j}^{{\rm{EXON}}}$, ${G}_{j}^{3{\rm{UTR}}}$ and ${G}_{j}^{5{\rm{UTR}}}$, which each take the value 1 if the *j*th SNV mapped to the respective genic annotation and 0 otherwise. We also mapped index SNVs and null SNVs to ATAC-seq peaks from single-cell atlases of chromatin accessibility (CATLAS and DESCARTES) for: 222 cell types derived from 30 human adult and 15 human fetal tissues^25,26^; and 106 cell types derived from human brain^27^. We defined an indicator variable, *X*_*ij*_, that takes the value 1 if the *j*th SNV mapped to an ATAC-seq peak for the *i*th cell type and 0 otherwise.

Within each cluster, we modelled enrichment of T2D associations for ATAC-seq peaks in the *i*th cell type, after accounting for genic annotations, in a Firth bias-reduced logistic regression, given by

$${f}^{-1}\left({Y}_{j}\right)={\alpha }_{0}+{\alpha }_{{\rm{EXON}}}{G}_{j}^{{\rm{EXON}}}+{\alpha }_{3{\rm{UTR}}}{G}_{j}^{3{\rm{UTR}}}+{\alpha }_{5{\rm{UTR}}}{G}_{j}^{5{\rm{UTR}}}+{\theta }_{i}{X}_{ij},$$

where *f* is the logit link function. In this expression, *α*_0_ is an intercept, *α*_EXON_, *α*_3UTR_ and *α*_5UTR_ are log fold enrichments of genic annotations, and *θ*_*i*_ is the log fold enrichment of ATAC-seq peaks in the *i*th cell type. We conducted a test of enrichment of the *i*th cell type by comparing the deviances of models in which *θ*_*i*_ *=* 0 and *θ*_*i*_ is unconstrained. We identified cell types with significant evidence of enrichment (*P* < 0.00023, Bonferroni correction for 222 cell types in adult and fetal tissues; *P* < 0.00047, Bonferroni correction for 106 cell types in the brain). All models were fitted using the R package logistf (https://cran.r-project.org/package=logistf).

### Contribution of each axis of genetic variation to ancestry-correlated heterogeneity

For each index SNV, we calculated a *z*-score (beta/SE) for association with each axis of variation by aligning the effect from the meta-regression model to the T2D risk allele. For each index SNV, we identified the axis of genetic variation with the strongest association (greatest magnitude *z*-score).

### Differences in ancestry-correlated heterogeneity between mechanistic clusters

We tested for differences in *z*-scores (beta/SE) for association of index SNVs in each cluster with the *i*th axis of genetic variation by comparing two linear models by ANOVA: (i) ${f}^{-1}\left({Z}_{ij}\right)={\tau }_{0i}$; and (ii) ${f}^{-1}\left({Z}_{ij}\right)={\sum }_{k}{\tau }_{ki}{C}_{jk}$. In these expressions: *f* is the identity link function; *Z*_*ij*_ is the *z*-score for the *j*th index SNV; *C*_*jk*_ is an indicator variable that takes the value 1 if the *j*th index SNV was assigned to the *k*th cluster and 0 otherwise; and *τ*_0*i*_ and *τ*_*ki*_ are regression coefficients. Regression models were fitted using the glm function in R.

### Effect of BMI on ancestry-correlated and residual heterogeneity in allelic effects between GWASs

For each index SNV, we aggregated inverse-variance weighted allelic effects across GWASs by linear regression, implemented in MR-MEGA^10^, including as covariates: (i) three axes of genetic variation; (ii) mean BMI in controls; and (iii) mean BMI in T2D cases. After adjustment for BMI, we tested for: (i) ancestry-correlated allelic effect heterogeneity between GWASs; and (ii) residual allelic effect heterogeneity between GWASs. After adjustment, as outlined above, we re-assessed: (i) the contribution of each axis of genetic variation to ancestry-correlated heterogeneity; and (ii) the difference in ancestry-correlated heterogeneity between mechanistic clusters.

### Cluster-specific partitioned PS analyses of vascular outcomes and age of T2D onset

We tested for association of cluster-specific components of the partitioned PS and an overall PS with T2D-related macrovascular outcomes (CAD, ischaemic stroke and peripheral artery disease), microvascular complications (ESDN and proliferative diabetic retinopathy) and age of T2D onset in participants from the All of Us Research Program (AoURP; AFA, EUR and HIS ancestry groups), Biobank Japan (BBJ; EAS ancestry group), and Genes & Health (G&H; SAS ancestry group). Cohort descriptions and details of sequencing and genotyping, quality control and phenotype derivation are provided in the Supplementary Methods.

We conducted analyses separately for each ancestry group in AoURP, BBJ and G&H. For each ancestry, we performed analyses for macrovascular outcomes using all individuals, irrespective of T2D status, and for microvascular complications in individuals with T2D only. For each analysis, we calculated the overall PS and cluster-specific partitioned PS for each individual, with each index SNV weighted by the allelic log-OR from the ancestry-specific meta-analyses. We did not include index SNVs with MAF < 1% in the PS. We also excluded index SNVs with poor imputation quality (*r*^2^ < 0.7) in BBJ and G&H, and those with extreme deviation from Hardy–Weinberg equilibrium (*P* < 10^−6^) in AoURP. We standardized the overall PS and each cluster-specific component of the partitioned PS to have mean zero and unit variance. We tested for association with each vascular outcome through generalized linear regression and with age of T2D onset through linear regression. For each outcome, we considered a model including the overall PS and then each cluster-specific component the partitioned PS adjusted for the overall PS. All association analyses were conducted using the glm function in R.

We adjusted association analyses with vascular outcomes for age, sex and the first 20 principal components. In BBJ, we also adjusted for recruitment phase and status of the registered common diseases (other than T2D) to account for ascertainment. We further adjusted analyses of macrovascular outcomes for T2D status. We also further adjusted analyses of microvascular complications for duration of T2D. In AoURP, we defined age as age at last hospital visit. In BBJ, we defined age as age at first record. In G&H, we defined age as age at diagnosis for T2D cases and age at last follow-up for controls. For CAD, we also conducted sensitivity analyses by including, as an additional covariate, a CAD PS from the largest published multi-ancestry CAD GWAS^39^. The PS was constructed from index SNVs for 241 conditionally independent CAD associations, weighted by the multi-ancestry allelic log-OR (ancestry-specific effects were not available), and standardized to have mean zero and unit variance. We adjusted association analyses with age of T2D onset for sex and the first 20 principal components. In BBJ, we also adjusted for recruitment phase and status of the registered common diseases (other than T2D) to account for ascertainment.

For each outcome, we aggregated association summary statistics from each cluster-specific component of the partitioned PS and the overall PS across ancestries through random-effects meta-analyses. All meta-analyses were conducted using the R package meta (https://cran.r-project.org/package=meta).

### Cluster-specific partitioned PS analyses of clinical outcomes

We tested for association of cardiovascular and kidney-related clinical outcomes in EUR individuals with T2D in prospective GWASs from six clinical trials from the Thrombolysis in Myocardial Infarction (TIMI) Study Group (https://timi.org/). Trial descriptions and details of genotyping and quality control are provided in the Supplementary Methods.

Within each trial, we calculated the overall PS and cluster-specific components of the partitioned PS for each individual, with each index SNV weighted by the allelic log-OR from the European ancestry-specific meta-analysis. We standardized the overall PS and each cluster-specific component of the partitioned PS to have mean zero and unit variance. Data from the six trials were subsequently pooled, and we considered the following clinical outcomes in patients with T2D only: myocardial infarction, ischaemic stroke, cardiovascular death, hospitalization for heart failure, atrial fibrillation, acute limb ischaemia, peripheral revascularization, end-stage renal disease or renal death and albuminuria. We tested for association of each cluster-specific component of the partitioned PS with each clinical outcome under a Cox proportional hazards model, including age, sex, the first ten principal components and the overall PS as covariates. All association analyses were conducted using the coxph function with Efron ties handling from the R package survival (https://cran.r-project.org/package=survival).

### Ethics statement

Study-level ethics statements are provided in the Supplementary Note.

### Reporting summary

Further information on research design is available in the Nature Portfolio Reporting Summary linked to this article.

## Online content

Any methods, additional references, Nature Portfolio reporting summaries, source data, extended data, supplementary information, acknowledgements, peer review information; details of author contributions and competing interests; and statements of data and code availability are available at 10.1038/s41586-024-07019-6.

### Supplementary information

Supplementary Information Supplementary Note, including Supplementary Text, Methods, Acknowledgements and Funding, Ethics statements, lists of contributors and consortia members, and Supplementary Figs. 1–13.

Reporting Summary

Supplementary Tables This file contains Supplementary Tables 1–25

## Data Availability

Genome-wide association summary statistics from the multi-ancestry meta-analysis and ancestry-specific meta-analyses reported in this study are available through the DIAGRAM Consortium website (http://www.diagram-consortium.org/downloads.html).

## Code availability

Analyses were conducted using publicly available software: the UCSC LiftOver tool (https://genome.ucsc.edu/cgi-bin/hgLiftOver), MR-MEGA v.0.2 (https://genomics.ut.ee/en/tools), METAL v.2011-03-25 (https://genome.sph.umich.edu/wiki/METAL), PLINK v.1.9 (https://www.cog-genomics.org/plink/1.9/), Beagle 4.1 (https://faculty.washington.edu/browning/beagle/b4_1.html), SNPTEST v.2.5.6 (https://www.well.ox.ac.uk/~gav/snptest/), GWAMA v.2.2.2 (https://genomics.ut.ee/en/tools), EIGENSOFT v.7.2.1 (https://www.hsph.harvard.edu/alkes-price/software/), PLINK v.2.0 (https://www.cog-genomics.org/plink/2.0/), SHAPEIT4 (https://odelaneau.github.io/shapeit4/), Minimac4 (https://genome.sph.umich.edu/wiki/Minimac4), KING v.2.3 (https://www.kingrelatedness.com/) and EAGLE v.2.4 (https://alkesgroup.broadinstitute.org/Eagle/#Xeagle2). Analyses were also conducted using the following R packages: meta (https://cran.r-project.org/package=meta), ClustImpute (https://cran.r-project.org/package=ClustImpute), NbClust (https://cran.r-project.org/package=NbClust), factoextra (https://cran.r-project.org/package=factoextra) and logistf (https://cran.r-project.org/package=logistf).
