# Supplementary material for: Genetic drivers of heterogeneity in type 2 diabetes pathophysiology
Source: Nature. 2024 Feb 19;627(8003):347–57. doi: 10.1038/s41586-024-07019-6 (PMC10937372; doi:10.1038/s41586-024-07019-6)
Supplement: Supplementary file 2 — Reporting Summary [file 41586_2024_7019_MOESM2_ESM.pdf]

nature portfolio

Corresponding author(s): Andrew Morris, Konstantinos Hatzikotoulas, Benjamin Voight, Eleftheria Zeggini

Last updated by author(s): Nov 17, 2023

Reporting Summary

Nature Portfolio wishes to improve the reproducibility of the work that we publish. This form provides structure for consistency and transparency in reporting. For further information on Nature Portfolio policies, see our Editorial Policies and the Editorial Policy Checklist.

Statistics

For all statistical analyses, confirm that the following items are present in the figure legend, table legend, main text, or Methods section.

n/a	Confirmed	
☐	☒	The exact sample size (*n*) for each experimental group/condition, given as a discrete number and unit of measurement
☒	☐	A statement on whether measurements were taken from distinct samples or whether the same sample was measured repeatedly
☐	☒	The statistical test(s) used AND whether they are one- or two-sided<br>*Only common tests should be described solely by name; describe more complex techniques in the Methods section.*
☐	☒	A description of all covariates tested
☐	☒	A description of any assumptions or corrections, such as tests of normality and adjustment for multiple comparisons
☐	☒	A full description of the statistical parameters including central tendency (e.g. means) or other basic estimates (e.g. regression coefficient) AND variation (e.g. standard deviation) or associated estimates of uncertainty (e.g. confidence intervals)
☐	☒	For null hypothesis testing, the test statistic (e.g. *F*, *t*, *r*) with confidence intervals, effect sizes, degrees of freedom and *P* value noted<br>*Give P values as exact values whenever suitable.*
☒	☐	For Bayesian analysis, information on the choice of priors and Markov chain Monte Carlo settings
☒	☐	For hierarchical and complex designs, identification of the appropriate level for tests and full reporting of outcomes
☒	☐	Estimates of effect sizes (e.g. Cohen's *d*, Pearson's *r*), indicating how they were calculated

Our web collection on statistics for biologists contains articles on many of the points above.

Software and code

Policy information about availability of computer code

Data collection	No software were used for data collection.
Data analysis	Analyses were conducted using publicly available software: UCSC liftOver tool (https://genome.ucsc.edu/cgi-bin/hgLiftOver), MR-MEGA v0.2 (https://genomics.ut.ee/en/tools), METAL v2011-03-25 (https://genome.sph.umich.edu/wiki/METAL), PLINKv1.9 (https://www.cog-genomics.org/plink/1.9/), Beagle 4.1 (https://faculty.washington.edu/browning/beagle/b4_1.html), SNPTEST v2.5.6 (https://www.well.ox.ac.uk/~gav/snptest/), GWAMA v2.2.2 (https://genomics.ut.ee/en/tools), EIGENSOFT v7.2.1 (https://www.hsph.harvard.edu/alkes-price/software/), PLINKv2.0 (https://www.cog-genomics.org/plink/2.0/), SHAPEIT4 (https://odelaneau.github.io/shapeit4/), Minimac4 (https://genome.sph.umich.edu/wiki/Minimac4), KING v2.3 (https://www.kingrelatedness.com/), and EAGLE v2.4 (https://alkesgroup.broadinstitute.org/Eagle/#Xeagle2). Analyses were also conducted using the following R packages: meta (https://cran.r-project.org/package=meta), ClustImpute (https://cran.r-project.org/package=ClustImpute), NbClust (https://cran.r-project.org/package=NbClust), factoextra (https://cran.r-project.org/package=factoextra), and logistf (https://cran.r-project.org/package=logistf).

For manuscripts utilizing custom algorithms or software that are central to the research but not yet described in published literature, software must be made available to editors and reviewers. We strongly encourage code deposition in a community repository (e.g. GitHub). See the Nature Portfolio guidelines for submitting code & software for further information.

Data

Policy information about availability of data

All manuscripts must include a data availability statement. This statement should provide the following information, where applicable:

- Accession codes, unique identifiers, or web links for publicly available datasets
- A description of any restrictions on data availability
- For clinical datasets or third party data, please ensure that the statement adheres to our policy

Data availability. Genome-wide association summary statistics from the multi-ancestry meta-analysis and ancestry-specific meta-analyses reported in this study are available through the DIAGRAM Consortium website (http://www.diagram-consortium.org/downloads.html).

Human research participants

Policy information about studies involving human research participants and Sex and Gender in Research.

Reporting on sex and gender	The numbers of males and females for each contributing study are reported in Supplementary Table 3. Sex-stratified analyses were not conducted.
Population characteristics	Characteristics are presented for each contributing study in Supplementary Table 3.
Recruitment	Ascertainment of type 2 diabetes cases and controls for each contributing study are presented in Supplementary Table 1.
Ethics oversight	All human research was approved within each contributing study by the relevant institutional review boards and conducted according to the Declaration of Helsinki. All participants provided written informed consent. Ethics statements from each contributing study are provided in the Supplementary Note.

Note that full information on the approval of the study protocol must also be provided in the manuscript.

Field-specific reporting

Please select the one below that is the best fit for your research. If you are not sure, read the appropriate sections before making your selection.

☒ Life sciences ☐ Behavioural & social sciences ☐ Ecological, evolutionary & environmental sciences

For a reference copy of the document with all sections, see nature.com/documents/nr-reporting-summary-flat.pdf

Life sciences study design

All studies must disclose on these points even when the disclosure is negative.

Sample size	Our discovery GWAS meta-analysis and polygenic score test GWAS brought together the largest sample size of type 2 diabetes cases and (population) controls that was available to the Type 2 Diabetes Global Genomics Initiative.<br><br>Discovery GWAS meta-analysis. With our sample size of 428,452 T2D cases and 2,107,149 controls, at a genome-wide significance threshold (p<5x10-8), under an additive genetic model of homogeneous effects across ancestry groups, we had ≥80% power to detect association of SNVs with MAF ≥5% and OR ≥1.035 or MAF ≥0.5% and OR ≥1.107.<br><br>Polygenic score test GWAS. We aggregated 30,288 T2D cases and 249,264 controls from the All of Us Research Program, Biobank Japan, and Genes & Health, who were not included as part of the discovery meta-analysis. We also consider 29,827 individuals with T2D from six clinical trials from the Thrombolysis in Myocardial Infarction (TIMI) Study Group.
Data exclusions	Within each contributing study, individuals were excluded on the basis of well-established individual and variant quality control (QC) procedures to remove poor quality genotypes, samples and SNVs. These QC procedures are described in Supplementary Table 3 for each study.
Replication	We did not conduct a formal replication analysis since we had already brought together all GWAS data available to the Type 2 Diabetes Global Genomics Initiative. The polygenic score test GWAS were not used to replicate association signals from the discovery meta-analysis because sample overlap can lead to increased false positive error rates in polygenic score analyses. All reported association signals from the discovery meta-analysis were checked to confirm that effects were not driven by false positives in single studies.
Randomization	Randomization was not performed. Within each study, covariates were adjusted for to account for potential confounding. Covariate adjustments are reported in Supplementary Table 3.
Blinding	Group allocation was not relevant to this study, so blinding was not necessary.

Reporting for specific materials, systems and methods

We require information from authors about some types of materials, experimental systems and methods used in many studies. Here, indicate whether each material, system or method listed is relevant to your study. If you are not sure if a list item applies to your research, read the appropriate section before selecting a response.

Materials & experimental systems

n/a	Involved in the study
☒	☐ Antibodies
☒	☐ Eukaryotic cell lines
☒	☐ Palaeontology and archaeology
☒	☐ Animals and other organisms
☒	☐ Clinical data
☒	☐ Dual use research of concern

Methods

n/a	Involved in the study
☒	☐ ChIP-seq
☒	☐ Flow cytometry
☒	☐ MRI-based neuroimaging
